# *Lactiplantibacillus plantarum* AS21 Attenuates Sex‐Biased Glucose Intolerance in Aging Mice via Gut Mucosal Microbiota‐Derived Citrulline

**DOI:** 10.1111/acel.70662

**Published:** 2026-08-13

**Authors:** Mengya Jia, Mengyan Chen, Hu Chen, Huiling Zhang, Ruixue Dou, Chaorui Wang, Muhammad Ishaq, Xiaoying Wei, Zitong Ding, Xusheng Guo

**Affiliations:** ^1^ School of Life Sciences Lanzhou University Lanzhou Gansu Province China; ^2^ Probiotics and Life Health Institute Lanzhou University Lanzhou Gansu Province China

**Keywords:** aging, citrulline, glucose intolerance, gut mucosal microbiota, homeostasis, probiotic, sex‐difference

## Abstract

Aging‐related metabolic decline, particularly impaired glucose tolerance, represents a growing challenge in aging populations. Probiotics are promising modulators of host metabolism; however, how sex differences influence their efficacy in mitigating metabolic aging remains poorly understood. Here, we administered *Lactiplantibacillus plantarum* AS21 to naturally aged male and female mice for 6 months. We observed a pronounced sexual dimorphism in metabolic decline, with aged males exhibiting more severe glucose intolerance than females. Concurrently, circulating citrulline levels were depleted in males but elevated in females. AS21 intervention significantly ameliorated glucose intolerance in aged males, while exerting minimal effects on metabolically stable females. Microbiota analysis revealed that AS21 reshaped the mucosa‐associated microbial communities in males, an effect accompanied by increased abundance of microbial citrulline biosynthesis genes (*argF*, *argI*) and restoration of circulating citrulline. In contrast, females maintained stable metabolic and citrulline profiles alongside limited microbial alterations. Furthermore, direct citrulline supplementation recapitulated these metabolic benefits, improving glucose tolerance, enhancing glucose‐stimulated insulin secretion (GSIS), and stimulating glucagon‐like peptide‐1 (GLP‐1) release. Collectively, these findings demonstrate that AS21 alleviates age‐related glucose dysregulation in male mice—a benefit closely associated with the restoration of microbiota‐driven citrulline metabolism. This highlights its potential as a sex‐specific, microbiota‐targeted strategy for managing metabolic aging.

## Graphical abstract

Aging induces sex‐differential glucose intolerance, with male‐specific citrulline depletion. *Lactiplantibacillus plantarum* AS21 elevates microbiota‐derived citrulline metabolism and enhances GLP‐1 secretion, improving glucose homeostasis.

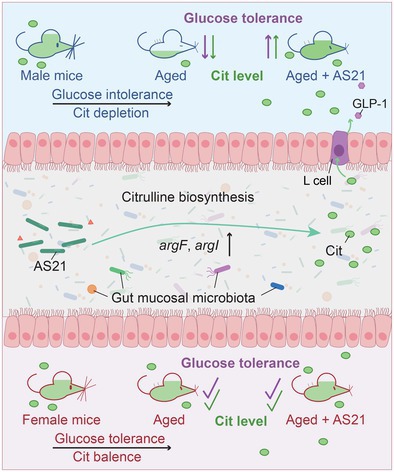

## 1 Introduction

Maintaining metabolic resilience is essential for promoting healthy aging, with disruption of glucose homeostasis representing a key hallmark of age‐associated metabolic decline. Prediabetes, an intermediate state between normoglycemia and type 2 diabetes (Birkenfeld and Mohan 2024), affects a substantial proportion of the global population and constitutes a critical window for early intervention due to its potential reversibility (Chakkalakal et al. 2024; International Diabetes Federation 2025). However, current therapeutic strategies are largely designed for overt disease, leaving a significant gap in targeted interventions for individuals at the pre‐disease stage beyond lifestyle modification. Importantly, biological sex is increasingly recognized as a fundamental variable in aging (Zhang et al. 2021), shaping divergent trajectories of metabolic dysfunction, including differences in glucose regulation and susceptibility to prediabetes (Ji et al. 2022). Despite this, most intervention strategies do not explicitly account for sex‐specific metabolic vulnerability (Kroemer et al. 2025), highlighting a critical unmet need for precision approaches that integrate aging and sex biology.

The gut microbiota is now widely established as a key regulator of host metabolic homeostasis, undergoing dynamic compositional and functional remodeling during aging (Sun et al. 2023). Targeting the gut microbiota has therefore emerged as a promising strategy to mitigate age‐associated metabolic dysfunction. Among these approaches, probiotics have gained particular attention as tractable and potentially translatable interventions capable of modulating host metabolism through microbiota‐dependent mechanisms. Accumulating evidence indicates that probiotics can improve metabolic outcomes by alleviating β‐cell endoplasmic reticulum stress (Sargin et al. 2022), enhancing antioxidant defenses (Soleimani et al. 2017), regulating lipid‐sensing pathways (Bauer et al. 2018), and modulating enteroendocrine signaling (Wang et al. 2025).

Despite these advances, the mechanistic basis underlying probiotic‐mediated metabolic benefits remains incompletely understood. Most studies have focused on classical microbiota‐derived metabolites, such as short‐chain fatty acids (Huang et al. 2025) and bile acids (Zheng et al. 2021), whereas the microbial regulation of amino acid metabolism has only recently emerged as an additional and underexplored layer of host–microbe cross‐talk. Notably, alterations in microbial amino acid metabolic capacity can profoundly reshape systemic metabolic phenotypes (Li et al. 2024). However, whether probiotic interventions can modulate microbiota‐dependent amino acid metabolism, and how sexual dimorphism influences these processes during metabolic aging, remains largely unknown.

Amino acid dysregulation is a prominent feature of metabolic aging and impaired glucose homeostasis, often characterized by elevated branched‐chain amino acids and reduced levels of specific non‐essential amino acids (Qin et al. 2025; Zhu and Goodarzi 2020). Citrulline, a non‐proteinogenic amino acid primarily synthesized in the small intestine, plays essential roles in nitrogen balance, redox regulation, and intestinal integrity (Mao et al. 2022; Papadia et al. 2018; Xie et al. 2025). Beyond these functions, accumulating evidence suggests a potential role for citrulline in glucose metabolism (Bagheripour et al. 2023). Intriguingly, citrulline levels consistently decline during aging in mouse models and certain human cell populations (Xie et al. 2025). However, the physiological significance of this decline, particularly in the context of sex‐specific metabolic regulation, remains poorly defined.

Here, we identify a previously unrecognized, sex‐specific decline in circulating citrulline that is closely associated with impaired glucose tolerance in aged male mice. We further demonstrate that intervention with the probiotic *Lactiplantibacillus plantarum* AS21 (Li et al. 2022) exerts pronounced sex‐dependent metabolic effects in naturally aged mice. Specifically, AS21 ameliorates glucose intolerance in aged males, an effect accompanied by an enhanced microbiota‐associated capacity for citrulline biosynthesis and a partial restoration of circulating citrulline levels. In contrast, female mice maintain relatively stable glucose homeostasis and exhibit minimal changes in citrulline levels. Collectively, these findings highlight a potential mechanistic link between microbiota‐associated citrulline metabolism and sex‐biased glucose intolerance during aging, supporting the development of microbiota‐targeted probiotic strategies to mitigate sex‐specific, age‐related impairments in glucose homeostasis.

## 2 Materials and Methods

### 2.1 Probiotic Strain and Preparation of Probiotic Powder

AS21 was originally isolated from traditional Tibetan yak yogurt. The strain is preserved in the Qinghai–Tibet Plateau Lactic Acid Bacteria Germplasm Bank and deposited in the China General Microbiological Culture Collection Center (CGMCC No. 24968). To prepare the probiotic powder, bacterial cells were harvested, freeze‐dried, and ground into powder (see Supplementary Methods). Viable bacterial counts were determined by standard plate counting and expressed as colony‐forming units per gram (CFU/g).

### 2.2 Animals and Experimental Design

All animal procedures were approved by the Institutional Animal Care and Use Committee of Lanzhou University (Approval No. EAF2024027). Specific pathogen‐free C57BL/6J mice were housed under standard laboratory conditions (12‐h light/dark, 25°C ± 2°C, 50% ± 5% relative humidity) with ad libitum access to chow and water.

#### 2.2.1 Natural Aging Model

Male and female C57BL/6J mice aged 14 and 18 months (*n* = 8 per sex/group) were utilized. The 18‐month‐old cohorts received either AS21 (1 × 10^9^ CFU suspended in 200 μL PBS) or PBS vehicle control via oral gavage for a duration of 6 months. The probiotic was administered daily for the first week and every 3 days thereafter. Additional cohorts of 3‐ and 14‐month‐old mice were included for baseline comparisons. To minimize the confounding effects of early‐life environmental variations, the baseline cohorts were maintained under identical environmental conditions as the aged groups and were acclimated for 6 weeks prior to sample collection.

#### 2.2.2 Prediabetes Model

Fifteen‐month‐old male mice (*n* = 10/group) were fed either a normal chow diet (NC; D12450H) or a high‐fat diet (HFD; D12451). Either AS21 (1 × 10^9^ CFU in 200 μL PBS) or PBS vehicle was administered daily via oral gavage. To induce glucose impairment, a modified streptozotocin (STZ) protocol was employed: STZ was freshly dissolved in 0.1 M sodium citrate buffer (pH 4.5) and administered via intraperitoneal injection on experimental Days 59, 61, and 72 at doses of 20, 20, and 10 mg/kg body weight, respectively. Probiotic and vehicle interventions were temporarily suspended during the STZ treatment window and resumed 72 h following the final STZ injection.

### 2.3 Glucose Homeostasis Tests

Blood samples were obtained via gentle tail tip pricking, and circulating glucose levels were measured using a handheld glucometer (ACCU‐CHEK Instant, Roche). During the testing periods, mice had ad libitum access to water, with food access restored immediately upon test completion.

#### 2.3.1 Oral Glucose Tolerance Test (OGTT)

Mice were transferred to clean cages and subjected to a 6‐h fast. Baseline fasting blood glucose (0 min) and body weight were recorded. Mice were then administered a dextrose load (2 g/kg body weight; prepared as 20% solution in sterile ddH_2_O) via oral gavage. Blood glucose levels were subsequently measured at 15, 30, 60, 90, and 120 min post‐gavage.

#### 2.3.2 Insulin Tolerance Test (ITT)

Mice were fasted for 4 h, after which baseline blood glucose and body weight were recorded. Insulin was then administered intraperitoneally at 0.6 U/kg (males) or 0.4 U/kg (females) for the natural aging cohorts, and 1.0 U/kg for the prediabetic model. Blood glucose was measured at 15, 30, 60, 90, and 120 min post‐injection, with an additional measurement at 150 min in the prediabetic model.

#### 2.3.3 Glucose‐Stimulated Insulin Secretion

Glucose‐stimulated insulin secretion (GSIS) was evaluated using plasma samples collected concurrently with the OGTT procedure. Blood was collected from the tail tip directly into EDTA‐coated tubes at 0, 15, and 30 min following the dextrose gavage. The samples were centrifuged at 3000 rpm for 10 min to isolate the plasma fraction. Extracted plasma samples were immediately stored at −80°C until downstream analysis. Plasma insulin concentrations were quantitatively determined using a high‐sensitivity mouse insulin ELISA kit (#90080, Crystal Chem).

### 2.4 Biochemical and Hormonal Measurements

Serum levels of inflammatory cytokines and metabolic hormones, including IL‐6 (Mlbio, ml063159), IFN‐γ (Mlbio, ml002277), IL‐10 (Mlbio, ml037873), TNF‐α (Mlbio, ml002095), IL‐1β (Mlbio, ml098416), and insulin (Mlbio, ml001983), were measured at the experimental endpoint using ELISA kits. In the prediabetes model, circulating glucagon‐like peptide‐1 (GLP‐1) levels were quantified using plasma samples collected for GSIS via an ELISA kit (Jonln, JL11122). Hepatic oxidative stress parameters, encompassing superoxide dismutase (SOD; Nanjing Jiancheng Bioengineering Institute, A001‐3) activity and malondialdehyde (MDA; Nanjing Jiancheng Bioengineering Institute, A003‐1) levels, were determined using biochemical assay kits. Serum biochemical indicators, including aspartate aminotransferase (AST), total bilirubin (TB), and blood urea nitrogen (BUN), were measured using a V‐200 automated biochemical analyzer (URIT, China).

### 2.5 Untargeted Metabolomic Profiling

Serum samples obtained from the natural aging model were subjected to untargeted metabolomic analysis (Wekemo Tech, Shenzhen, China). UHPLC–MS/MS analysis was performed using a Vanquish UHPLC system coupled to an Orbitrap Q Exactive HF mass spectrometer (Thermo Fisher Scientific, Germany). Raw data were processed utilizing Compound Discoverer 3.3 (Thermo Fisher Scientific) for peak alignment, peak detection, and quantification. Metabolites were identified by matching against the mzCloud, mzVault, and MassList databases, and were further annotated using the KEGG and HMDB databases. Detailed instrumental parameters are provided in Supplementary Methods. Differentially abundant metabolites were determined employing a Student's *t*‐test with false discovery rate (FDR) correction. Metabolites satisfying the criteria of |log_2_ fold change| > 0.58 and FDR‐adjusted *p* < 0.05 were considered significantly altered.

### 2.6 Metagenomic Sequencing and Analysis of Colonic Mucosal Microbiota

Colon tissues were opened longitudinally and gently rinsed with PBS, after which the mucosal layer was carefully scraped off for microbial genomic DNA extraction. Sequencing libraries were constructed using the NEBNext Ultra DNA Library Prep Kit and sequenced on an Illumina NovaSeq 6000 platform to generate approximately 10 Gb paired‐end reads (150 bp) per sample. Metagenomic reads were quality‐filtered, host sequences removed, and taxonomic and functional profiling performed using standard bioinformatic pipelines. Detailed procedures are described in Supplementary Methods. Alpha diversity was evaluated using Chao1, Observed Features, Shannon, and Simpson indices. Beta diversity was assessed based on Bray–Curtis distances and visualized via principal coordinate analysis (PCoA), and statistical significance was determined utilizing PERMANOVA. Differentially abundant taxa between groups were identified using the Wilcoxon rank‐sum test.

### 2.7 Metagenomic Cluster Analysis and Phylogenetic Tree Construction

Metagenome‐assembled genomes (MAGs) were reconstructed from colonic mucosal metagenomic sequencing data, following the initial assembly procedures described in Section 2.6. MAG‐derived ArgF sequences were compared with homologs retrieved from the NCBI nr database. Phylogenetic analysis was performed using the Neighbor‐Joining method in MEGA12 with 1000 bootstrap replicates. The related protein sequences are listed in Table S3.

### 2.8 In Vitro Co‐Culture of Mucosa‐Associated Microbiota

In vitro co‐culture experiments were performed as previously described, with minor modifications (Wang et al. 2024). Briefly, after removing the luminal contents, colonic tissues were rinsed with sterile saline and homogenized (1:9, w/v). The resulting supernatant was inoculated (1%, v/v) into brain heart infusion (BHI) medium. These cultures were incubated anaerobically at 37°C for 24 h to specifically enrich the mucosa‐associated microbiota.

AS21 was cultured in MRS medium and subsequently prepared into four distinct fractions: live bacteria, cell‐free culture supernatant, cell lysate, or heat‐killed bacterial suspensions. Each specific AS21 preparation (10%, v/v) was added to the enriched mucosal microbiota and co‐cultured under anaerobic conditions. Culture supernatants were collected at 24 h, whereas both supernatants and bacterial cell pellets were harvested at 48 h. In designated experimental groups, α‐ketoglutarate (AKG) was added to a final concentration of 1 mM.

### 2.9 Quantification of Metabolites in Co‐Culture Supernatants

Amino acid‐related metabolites were quantified via LC–MS/MS (LC‐Bio Technologies, Hangzhou, China) using an Agilent 1290 UHPLC system coupled to an Agilent 6545 Q‐TOF mass spectrometer (Agilent Technologies, USA). Metabolites were definitively identified based on accurate mass, retention time, and MS/MS fragmentation patterns and subsequently quantified relative to the internal standards.

### 2.10 Cell Culture

The mouse enteroendocrine STC‐1 cell line (BeNa Culture Collection, #350769) was utilized in this study. Cells were cultured in high‐glucose Dulbecco's modified Eagle's medium (DMEM) supplemented with 10% heat‐inactivated fetal bovine serum (FBS; Gibco, #A5256701) and 100 U/mL penicillin–streptomycin (Gibco, #15140122), unless otherwise specified. Cultures were maintained at 37°C in a humidified incubator containing a 5% CO_2_ atmosphere.

### 2.11 GLP‐1 Secretion and Production Analysis

For GLP‐1 secretion assays, STC‐1 cells were seeded and grown to 70%–80% confluency and subjected to serum deprivation for 2 h in DMEM containing 0.5% FBS. Subsequently, the cells were incubated in serum‐ and glucose‐free DMEM supplemented with either 500 μM citrulline (Beyotime, ST1480) or 10 mM glucose in the presence of 3 μM sitagliptin (MedChemExpress, MK‐0431) for 30 min. The culture supernatants were then collected, and GLP‐1 concentrations were quantified using an ELISA kit (Jonln, JL11122).

For GLP‐1 production analysis, cells were seeded and subjected to corresponding stimulatory conditions (500 μM citrulline or 10 mM glucose) in DMEM containing 0.5% FBS and 5.5 mM glucose for 24 h. Total RNA was extracted using TRIzol reagent for downstream gene expression analysis. In parallel, identically treated cells were processed for immunofluorescence staining, as described below.

### 2.12 Comet Assay

DNA damage was assessed using a comet assay kit (Beyotime, C2041M). STC‐1 cells were treated with 500 μM citrulline and then UVC irradiated (191 mJ/cm^2^). After single‐cell gel electrophoresis, DNA comets were stained with DAPI and imaged with an Axio Observer 7 microscope (ZEISS). At least 100 cells per experimental condition were analyzed using the OpenComet plugin for ImageJ (v1.54r).

### 2.13 Immunofluorescence Staining

Following the designated treatments, STC‐1 cells were fixed, permeabilized, and blocked. The samples were then incubated overnight at 4°C with a primary antibody against GLP‐1 (Affinity, AF0166), followed by a 1‐h incubation at room temperature with a secondary antibody (Affinity, S0006). Cellular nuclei were counterstained with DAPI. Fluorescence images were captured with an Axio Observer 7 microscope (ZEISS) and further processed with ZEN 3.1 and ImageJ (v1.54r).

### 2.14 Gene Expression Analysis

Total RNA was isolated from cells using an RNA extraction kit (YEASEN, 10606ES60) and subsequently reverse‐transcribed into cDNA using a targeted synthesis kit (YEASEN, 11141ES). Quantitative real‐time PCR was conducted utilizing a SYBR Green master mix (YEASEN, 11120ES). Relative gene expression levels were calculated by applying the standard 2^−ΔΔCt^ method, with target gene transcript levels normalized to the endogenous reference gene *Gapdh*. The specific primer sequences utilized are detailed in Table S2.

### 2.15 Quantification of Citrulline

Serum citrulline was quantified using a commercial ELISA kit (Yuanju Bio, YJ6002548) for the natural aging cohorts, and by targeted LC–MS/MS with external calibration for the prediabetic model. Chromatographic separation was performed on an Agilent 1290 UHPLC system equipped with a Kinesis HILIC column (100 × 2.1 mm, 2.6 μm; Phenomenex). Mass spectrometric detection was used with an Agilent 6470 triple quadrupole mass spectrometer in multiple reaction monitoring mode.

### 2.16 Systematic Review of Clinical Studies on Citrulline and Glycemic Outcomes

A systematic literature search was performed across the PubMed and Web of Science Core Collection databases (2015–2026) to identify clinical studies evaluating the effects of citrulline on glycemic outcomes. Study selection was conducted in accordance with the Preferred Reporting Items for Systematic Reviews and Meta‐Analyses (PRISMA) 2020 guidelines (Page et al. 2021). Detailed procedures are described in Supplementary Methods. Five clinical studies met the inclusion criteria, the details of which are summarized in Table S1.

### 2.17 Statistical Analysis

Data are expressed as the mean ± standard error of the mean (SEM). Statistical analyses were conducted using GraphPad Prism (version 9.5). Depending on experimental design and data distribution, significance was evaluated using unpaired Student's *t*‐test, one‐way or two‐way ANOVA, or appropriate non‐parametric alternatives (Mann–Whitney *U* test, the Kruskal–Wallis test). Correlations were assessed using Spearman's rank correlation coefficient. A *p*‐value < 0.05 was considered statistically significant.

For OGTT and ITT in the natural aging model, data from male and female cohorts were analyzed independently at each time point using ordinary one‐way ANOVA or the Kruskal–Wallis test, as appropriate. Overall *p*‐value summaries for each time point are presented in the main figures, with detailed multiple‐comparison results in the Supporting Information: Figures. Each experimental group was compared against the corresponding 24‐month‐old (24 m) control group of the same sex using Dunnett's or Dunn's multiple comparison test.

## 3 Results

### 3.1 AS21 Maintains Glucose Homeostasis During Aging in Male Mice

**FIGURE 1:**
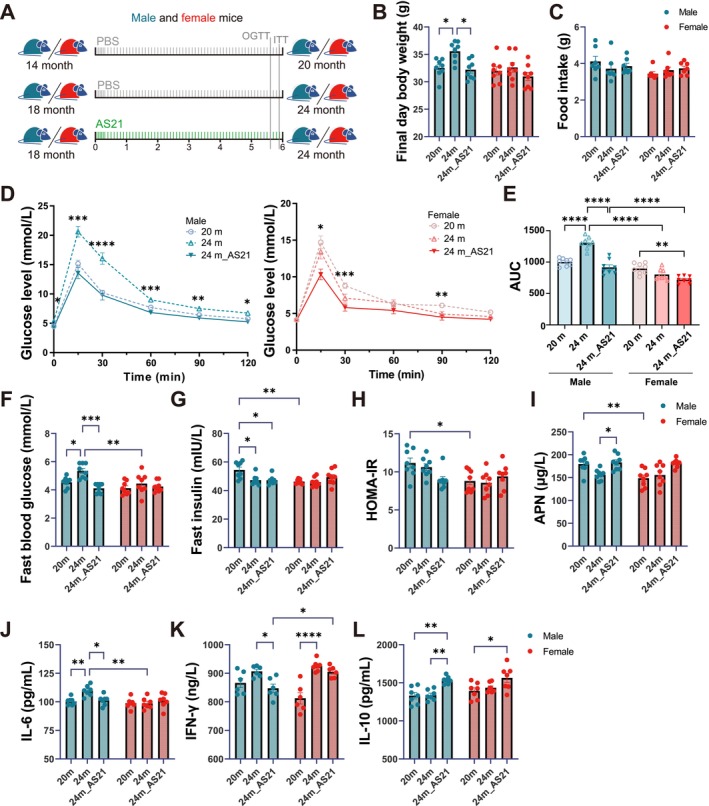
AS21 intervention alleviates age‐associated glucose intolerance and metabolic inflammation in male mice. (A) Experimental design of the natural aging mouse model. Naturally aged mice (14 and 18 months old at the start of intervention) were maintained for an additional 6 months. AS21 (1 × 10^9^ CFU per mouse) was administered by gavage daily during the first week, followed by administrated three times per week thereafter. (B) Body weight at the end of the intervention period. (C) Average food intake during the final week of the intervention. (D) Oral glucose tolerance test (OGTT) in male and female mice. (E) Area under the curve (AUC) for the OGTT. (F) Fasting blood glucose levels. (G) Fasting serum insulin levels. (H) HOMA‐IR index. (I) Serum adiponectin (APN) levels. (J–L) Serum inflammatory cytokines, including IL‐6, IFN‐γ, and IL‐10. Statistics: Two‐way ANOVA was applied for comparisons among the six experimental groups unless otherwise specified. For the OGTT analyses, data from male and female mice were analyzed separately at each time point using ordinary one‐way ANOVA or the Kruskal–Wallis test, as dictated by data normality. Overall *p*‐value summaries for each time point are presented in (D). ns, *p* > 0.05; *p < 0.05; **p < 0.01; ***p < 0.001; ****p < 0.0001. Data points represent individual biological replicates. APN, adiponectin; AS21, *Lactiplantibacillus plantarum* AS21; AUC, area under the curve; HOMA‐IR, homeostatic model assessment of insulin resistance; IFN‐γ, interferon‐γ; IL‐6, interleukin‐6; IL‐10, interleukin‐10; OGTT, oral glucose tolerance test; PBS, phosphate buffer saline.

To assess the long‐term metabolic impacts of probiotic intervention during natural aging, 18‐month‐old male and female mice received AS21 orally for 6 months (Figure 1A). An OGTT revealed severely impaired glucose tolerance in 24‐month‐old males relative to 20‐month‐old controls, whereas females showed minimal age‐related differences between these time points (Figure 1D,E and Figure S2). Conversely, ITT detected no substantial changes between the 20‐ and 24‐month‐old cohorts (Figures S1I,J and S3). Notably, long‐term AS21 intervention effectively preserved glucose homeostasis exclusively in aged males (Figure 1D,E and Figure S2). Consistent with this glucose intolerance, 24‐month‐old male mice exhibited elevated fasting blood glucose and serum IL‐6, alongside decreased fasting insulin, compared to 20‐month‐old counterparts (Figure 1F–L). Importantly, AS21 treatment in aged males significantly reduced fasting blood glucose, serum IL‐6 and IFN‐γ, while concurrently elevating serum adiponectin (APN) and IL‐10 levels. Furthermore, AS21 mitigated age‐associated body weight gain without altering food intake (Figure 1 and Figure S1). Collectively, these findings reveal a pronounced sexual dimorphism during natural aging, whereby male mice experience a more severe deterioration of glucose homeostasis and systemic inflammation than females. Crucially, long‐term AS21 intervention confers robust, sex‐specific protection against these age‐related metabolic and inflammatory impairments in males.

### 3.2 Sex‐Dimorphic Decline in Circulating Citrulline During Aging

**FIGURE 2:**
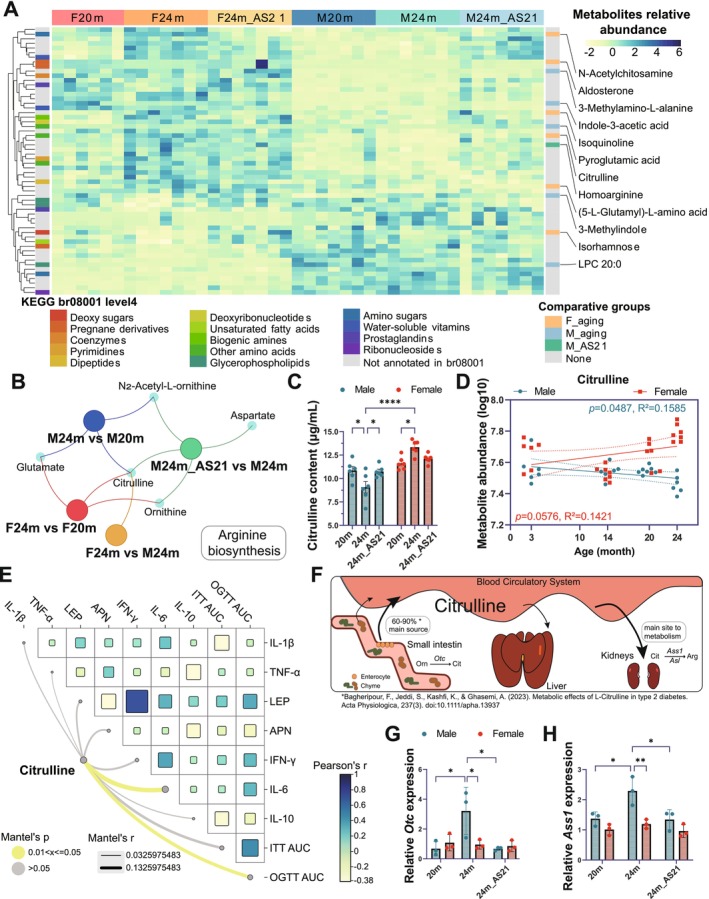
Sexually dimorphic decline in circulating citrulline during aging is associated with glucose intolerance and metabolic alterations in male mice. (A) Heatmap showing differentially abundant serum metabolites between F24m and M24m groups. Metabolites identified in aging‐, sex‐dependent‐, and probiotic‐treatment‐related comparisons are indicated. KEGG BRITE hierarchy annotations were assigned according to br08001. (B) Network Venn diagram illustrating overlapping differentially enriched metabolites within the arginine biosynthesis pathway across experimental comparisons. (C) Serum citrulline concentrations quantified by ELISA. (D) Linear regression analysis showing the association between circulating citrulline levels and age. (E) Mantel's correlation analysis integrating circulating citrulline with biochemical and metabolic parameters. Pearson correlation coefficients were used for the biochemical index matrix. (F) Schematic overview of citrulline biosynthesis pathways in mammals. (G) *Otc* mRNA expression in ileal tissue. (H) Renal *Ass1* mRNA expression. Statistics: Two‐way ANOVA was used for comparisons among six experimental groups unless otherwise specified. Linear regression analysis was applied in panel (D). Mantel's analysis was used in panel (E). *p < 0.05; **p < 0.01; ****p < 0.0001. Data points represent individual biological replicates. APN, adiponectin; F20m, 20‐month‐old female mice; F24m, 24‐month‐old female mice; F24m_AS21, 24‐month‐old female mice treated with AS21; IFN‐γ, interferon‐γ; IL‐1β, interleukin‐1β; IL‐10, interleukin‐10; IL‐6, interleukin‐6; ITT AUC, area under the curve for the intraperitoneal insulin tolerance test; LEP, leptin; M20m, 20‐month‐old male mice; M24m, 24‐month‐old male mice; M24m_AS21, 24‐month‐old male mice treated with AS21; OGTT AUC, area under the curve for oral glucose tolerance testTNF‐α, tumor necrosis factor‐α.

To elucidate the metabolic alterations underlying sex‐specific glucose homeostasis and AS21 efficacy during aging, we performed untargeted serum metabolomics following the 6‐month period. Pairwise comparisons identified four differentially abundant metabolite sets: 24FM (F24m vs. M24m), F_aging (F24m vs. F20m), M_aging (M24m vs. M20m), and M_AS21 (M24m_AS21 vs. M24m). Metabolites within the 24FM set are visualized in a heatmap, with corresponding alterations in the other comparison sets annotated via color bars (Figure 2A). Functional annotation based on KEGG br08001 level 4 classified metabolites with known biological functions. Notably, several metabolites—including N‐acetylchitosamine, aldosterone, 3‐methylamino‐L‐alanine, indole‐3‐acetic acid, isoquinoline, pyroglutamic acid, citrulline, homoarginine, (5‐L‐glutamyl)‐L‐amino acid, 3‐methylindole, isorhamnose, and LPC 20:0—exhibited altered abundances associated with female aging, male aging, or AS21 innovation in aged males. A comprehensive heatmap detailing the full metabolite names is provided in Figure S4.

KEGG over‐representation analysis revealed that differentially abundant metabolites were consistently enriched in the arginine biosynthesis pathway across all four comparison groups (Figure S4). Crucially, citrulline was the only differential metabolite within this pathway shared among all four groups (Figure 2B), highlighting it as a potential central node in sex‐ and aging‐associated metabolic remodeling. Subsequent ELISA validation confirmed these inter‐group differences and sexually dimorphic aging patterns of serum citrulline (Figure 2C). To substantiate this, we analyzed serum samples from mice across multiple adult stages. Both untargeted profiling and targeted quantification consistently demonstrated an age‐associated depletion of circulating citrulline in males, a trend absent in females (Figure 2D and Figure S5). Importantly, AS21 intervention significantly preserved circulating citrulline levels in aged male mice (Figure 2C). Correlation analyses revealed that citrulline levels were inversely associated with impaired glucose tolerance and elevated IL‐6 (Figure 2E and Figure S5), linking citrulline decline to metabolic and inflammatory dysfunction. Collectively, these findings demonstrate sex‐dependent changes in circulating citrulline during aging, associated with greater metabolic vulnerability in males. AS21 partially restores circulating citrulline in aged males, suggesting that altered citrulline metabolism may be linked to sex‐specific metabolic resilience.

### 3.3 Aged Male Mice Exhibit Increased Citrulline Turnover and Metabolic Vulnerability

Citrulline is a central intermediate in the citrulline‐arginine cycle, supporting nitric oxide production and arginine biosynthesis, while contributing to ammonia detoxification via the urea cycle (Papadia et al. 2018). In mammals, approximately 60%–90% of circulating citrulline derives from the small intestine, bypasses hepatic metabolism, and is primarily utilized by the kidney (figure 2f; Bagheripour et al. 2023). To investigate whether alterations in host citrulline synthesis and utilization contributed to the sex‐dependent changes in circulating citrulline, we examined ileum ornithine transcarbamylase (*Otc*) and renal argininosuccinate synthase 1 (*Ass1*) expression. Aged males showed relatively higher ileal *Otc* expression compared to groups with higher circulating citrulline levels (Figure 2G), suggesting a compensatory upregulation of intestinal synthesis. Concurrently, renal *Ass1* expression was elevated in middle‐aged and aged males (Figure 2H). Notably, males consistently displayed higher renal *Ass1* expression than females across all examined stages, though differences at 3 and 20 months fell short of statistical significance (Figure S5). Elevated *Ass1* expression is commonly interpreted as a renal compensatory response to heightened hepatic detoxification demands (Xia et al. 2025). Given this association, we assessed hepatic oxidative stress alongside liver and kidney functional indicators. Aged male mice displayed exacerbated hepatic oxidative stress, indicated by reduced SOD activity and elevated MDA accumulation (Figure S3D). Importantly, AS21 intervention significantly rescued SOD activity and reduced MDA levels in the livers of aged males. Concordantly, serum TB and BUN levels were significantly normalized by AS21 intervention (Figure S5E), with these protective effects being predominantly observed in males. Notably, AS21 did not alter citrulline content within ileal or colonic tissues (Figure S5F), indicating that host intestinal citrulline production remained unaffected and was unlikely to drive the observed fluctuations in the circulating citrulline pool.

### 3.4 AS21 Enhances Microbiota‐Associated Citrulline Synthesis Potential in a Sex‐Dependent Manner

Given the critical role of the gut microbiota in reshaping the host amino acid landscape (Li et al. 2024) and the spatial proximity of mucosal communities to host signaling interfaces (Song et al. 2023), we focused our profiling on the colonic mucosal microbiota. Metagenomic analysis revealed pronounced sex‐dependent divergence during aging. Aged male mice consistently exhibited higher α‐diversity, with significantly higher Shannon and Simpson indices at 24 months of age (Figure S6A). Concordantly, β‐diversity analysis revealed significant structural separation between the male and female microbial communities (Figure S6B), although UpSet analysis indicated approximately 31%–50% of microbial species were shared across all groups (Figure S6E). At the phylum level, the mucosal microbiota of aged mice was dominated by Campylobacterota, with 24‐month‐old females exhibiting a higher relative abundance of this phylum than males (Figure S6C). At the genus level, both aged male and female mice showed marked accumulation of *Chlamydia* and *Helicobacter*, with *Helicobacter* being more abundant in 24‐month‐old females (Figure S6D). Species‐level analysis further revealed an enrichment of *Helicobacter apodemus* in both sexes, whereas *Helicobacter bilis* accumulation was more pronounced in female mice (Figure S6D).

**FIGURE 3:**
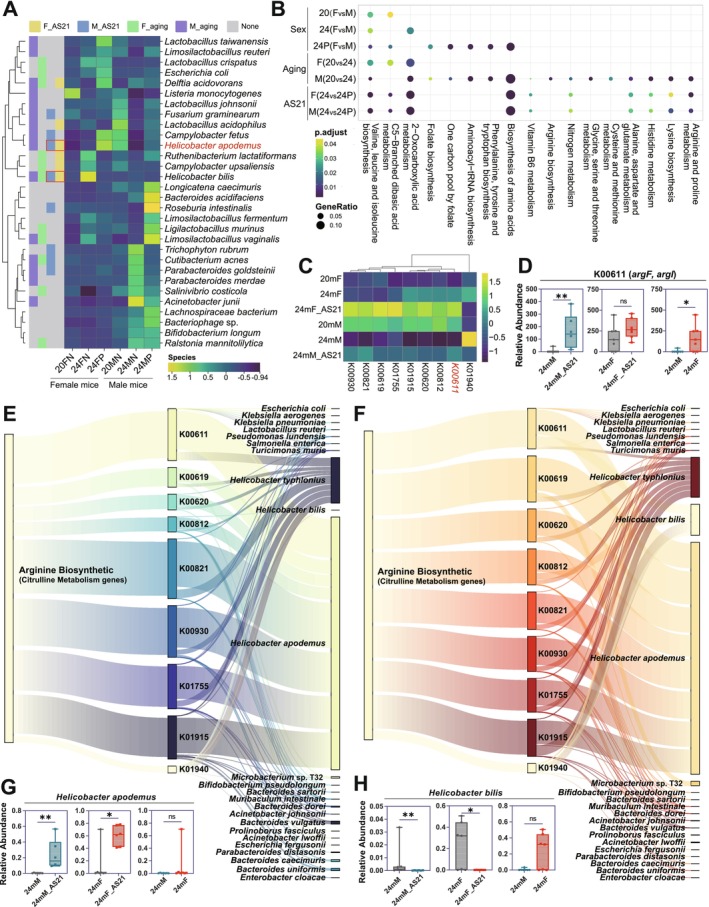
Sex‐dependent microbial remodeling by AS21 links to citrulline‐associated pathways. (A) Heatmap of differentially abundant mucosal microbiota species between female and male mice at 24 months of age. Color annotations indicate aging‐ and AS21 intervention‐associated comparisons. (B) Amino acid‐related pathways identified via KEGG ORA analysis of the mucosal microbiota. (C) Heatmap of citrulline metabolism‐related functional modules. (D) Relative abundance of K00611. (E, F) Species‐level source tracking of citrulline synthesis genes in the gut mucosal microbiome of male mice (E) and female mice (F). (G, H) Relative abundance of *Helicobacter apodemus* (G) and *Helicobacter billis* (H). Statistics: The two‐tailed Mann–Whitney *U* test was utilized for panels (D), (G) and (H). ns, *p* > 0.05; *p < 0.05; **p < 0.01. Data points represent individual biological replicates. In panel (A): F_AS21, differentially abundant species in 24‐month‐old female mice with AS21 intervention; M_AS21, differentially abundant species in 24‐month‐old male mice with AS21 intervention; F_aging, differentially abundant species between 20‐ and 24‐month‐old female mice; M_aging, differentially abundant species between 20‐ and 24‐month‐old male mice. In panel (B): 20(FvsM), 20‐month‐old females versus 20‐month‐old males; 24(FvsM), 24‐month‐old females versus 24‐month‐old males; 24P(FvsM), 24‐month‐old females with AS21 versus 24‐month‐old males with AS21; F(20vs24), 20‐month‐old versus 24‐month‐old females; M(20vs24), 20‐month‐old versus 24‐month‐old males; F(24vs24P), 24‐month‐old females versus 24‐month‐old females with AS21; M(24vs24P), 24‐month‐old males versus 24‐month‐old males with AS21.

To further characterize these sex‐specific microbial alterations in advanced aging, differential abundance analysis between 24‐month‐old male and female mice identified 30 microbial species (Figure 3A) and 21 genera (Figure S7A) that differed significantly. Based on DESeq2 analysis, aging‐associated (20m vs. 24m) and AS21‐responsive (24m vs. 24m_AS21) species were annotated with color bars (Figure 3A). Notably, aging‐affected and AS21‐responsive species showed limited overlap between males and females, suggesting a highly sexually dimorphic pattern of microbial remodeling. However, both *H. apodemus* and *H. bilis* were significantly altered by AS21 treatment in both sexes (Figure 3A). Functional profiling of the mucosal microbiota revealed that these sex‐ and age‐related microbial differences were significantly enriched in amino acid metabolism pathways. Notably, compared to females, male mice experienced more extensive age‐related disturbances in both microbial amino acid metabolism (Figure 3B) and global functional profiles (Figure S7B). Following probiotic intervention, both sexes exhibited concordant shifts in overall microbial functions, notably in arginine and proline metabolism, as well as general amino acid biosynthesis pathways (Figure 3B and Figure S7B).

In particular, probiotic treatment induced a generalized upregulation of KEGG orthologs (KOs) involved in citrulline synthesis within the arginine biosynthesis pathway (Figure 3C and Figure S8A). This effect was more pronounced in male mice, as exemplified by the key citrulline synthesis‐related genes K00611 (*argF*/*argI*) (Figure 3D). Gene traceability analysis revealed that the citrulline synthesis‐related genes were primarily contributed by *H. apodemus*. Compared to males, female mice exhibited a lower contribution from *H. apodemus* but a higher contribution from *H. bilis* (Figure 3E,F). Consistent with these taxonomic contributions, AS21 treatment significantly increased the relative abundance of *H. apodemus* while concurrently decreasing *H. bilis* in both sexes (Figure 3G,H). Therefore, there was no significant net increase in the relative abundance of mucosal K00611 (argF/argI) in females in spite of the expansion of *H. apodemus* following AS21 treatment (Figure 3D). In addition, the baseline of K00611 abundance in females was higher than in males (Figure 3D). These differential taxonomic shifts explain the sex‐dependent differences in K00611 abundance and the resulting citrulline biosynthetic capacity. Consequently, AS21 selectively enhanced citrulline biosynthesis‐related functional potential in the mucosal microbiota of aged males.

To provide robust genetic validation for these metagenomic observations, we reconstructed a metagenome‐assembled genome (MAG.H58.6) assigned to *H. apodemus* and successfully identified its resident citrulline synthesis genes (Figure S9). The ArgF protein encoded by MAG.H58.6 was identical to that of the *H. apodemus* reference sequence and clustered tightly with it in phylogenetic analysis (Figure S9C), confirming the presence of a conserved *argF* gene within the *H. apodemus* genome.

### 3.5 AS21 Enhances Microbial Citrulline Production Capacity In Vitro

**FIGURE 4:**
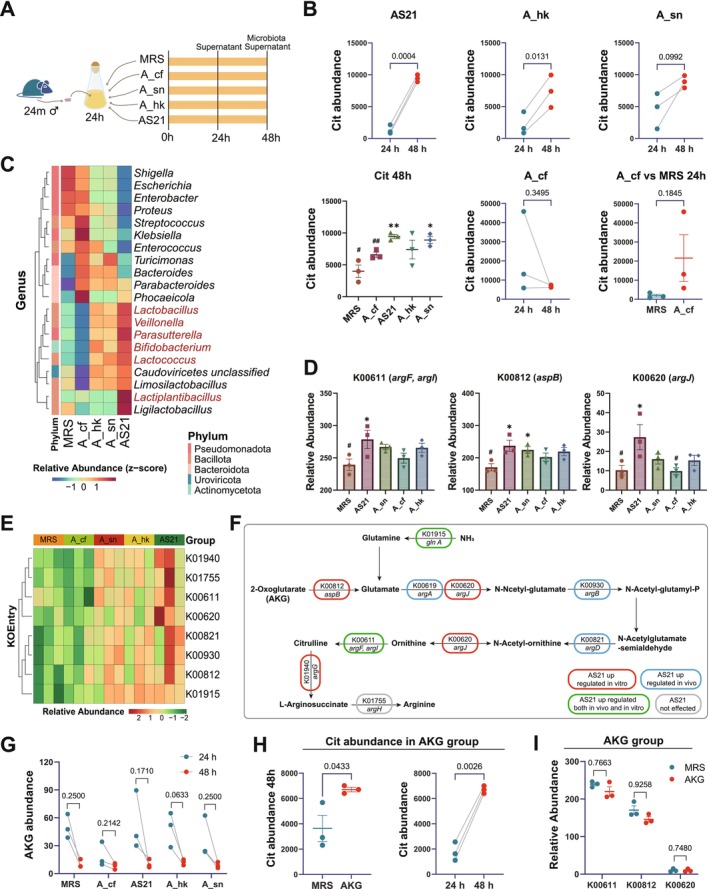
AS21 enhances microbial citrulline production capacity. (A) Schematic overview of the in vitro mucosal microbiota co‐culture experiment. (B) Citrulline concentrations in culture supernatants at indicated time points. (C) Heatmap of differentially abundant genera in co‐cultured treatments. (D) Relative abundance of representative citrulline biosynthesis genes in the co‐cultured microbiota. (E) Heatmap of KEGG orthologs involved in citrulline biosynthesis. (F) Schematic model of microbiome‐mediated citrulline synthesis pathways. (G) α‐ketoglutarate (AKG) levels across co‐cultured treatments. (H) Citrulline production following AKG supplementation. (I) Relative abundance of representative citrulline biosynthesis genes in AKG‐supplemented groups. Statistics: Paired two‐tailed *t*‐tests were utilized for comparisons between 24 and 48 h cultures. Unpaired *t*‐tests were used for two‐group comparisons. Multiple‐group comparisons were analyzed via ordinary one‐way ANOVA followed by Dunnett's post hoc test. Statistical significance is indicated in the figures. *p < 0.05, **p < 0.01 versus the MRS group; ^#^ *p* < 0.05, ^##^ *p* < 0.01 versus the AS21 group. Data points represent individual biological replicates. A_cf, cell‐free lysate of AS21; AKG, α‐ketoglutarate; A_hk, heat‐killed AS21; A_sn, AS21 culture supernatant; AS21, live AS21 strain; Cit, citrulline; MRS, de Man, Rogosa and Sharpe medium.

To determine whether AS21 influences microbial citrulline metabolism, we established an in vitro co‐culture system using cultures derived from colonic mucosa‐associated microbiota. Live AS21, cell‐free lysate (A_cf), culture supernatant (A_sn), heat‐killed bacteria (A_hk), or medium alone was added to the co‐culture system (Figure 4A). We then quantified citrulline levels and assessed shifts in both microbial community structure and citrulline biosynthesis genes. Treatments containing AS21 supernatant components—namely live AS21, A_sn, and A_hk—all led to a significant, time‐dependent increase in citrulline levels, reaching comparable accumulation by 48 h (Figure 4B). In contrast, the A_cf group, which lacked AS21 supernatant factors, exhibited a transient initial increase at 24 h but failed to sustain this trajectory; by 48 h, citrulline levels had declined significantly below those of the live AS21 group (Figure 4B). This indicates that soluble components secreted in the AS21 supernatant promote citrulline production by the microbiota.

Consistent with these metabolic dynamics, microbiome structural analysis revealed that communities co‐cultured with live AS21, A_sn, or A_hk shared a high compositional similarity, whereas those exposed to A_cf more closely resembled the medium control (Figure 4C and Figure S10A). Notably, AS21 enriched beneficial bacterial genera involved in short‐chain fatty acid metabolism, including *Lactobacillus*, *Veillonella*, *Bifidobacterium*, *Lactococcus*, and *Lactiplantibacillus*, along with the bile acid–modulating genus *Parasutterella* (Figure 4C). Functional analysis of citrulline synthesis genes revealed that the overall functional profile of the supernatant‐containing groups differed from that of the controls (Figure 4E). However, a significant increase in the abundance of key pathway genes (e.g., K00611 [*argF*, *argI*], K00812 [*aspB*], and K00620 [*argJ*]) was observed exclusively in co‐culture with live AS21, an increase that aligned closely with the expansion of specific source microorganisms (Figure 4D, Figures S10B and S11). These results indicate that while secreted metabolites can enhance baseline citrulline production, the active modulation of citrulline‐associated bacterial taxa and the targeted upregulation of functional genes require live AS21 cells.

Metabolomic profiling of the AS21 supernatant identified the secretion of upstream metabolites within the citrulline biosynthesis pathway, including glutamate, glutamine, and AKG (Figure S10G). Among these, AKG levels exhibited a non‐significant decreasing trend across all treatment groups over time (Figure 4G). Direct supplementation of AKG (1 mM) alone to the mucosal microbiota cultures was sufficient to significantly elevate extracellular citrulline production (Figure 4G). However, the robust upregulation of microbial citrulline biosynthesis genes observed with live AS21 treatment was not recapitulated by AKG addition alone.

Together, live AS21 enhances microbial citrulline production primarily by remodeling the community structure and enriching biosynthetic genes, an effect complemented by secreted endogenous metabolites, such as AKG, which serve as metabolic precursors.

### 3.6 AS21 and Citrulline Supplementation Improve Glucose Homeostasis in Prediabetic Mice

**FIGURE 5:**
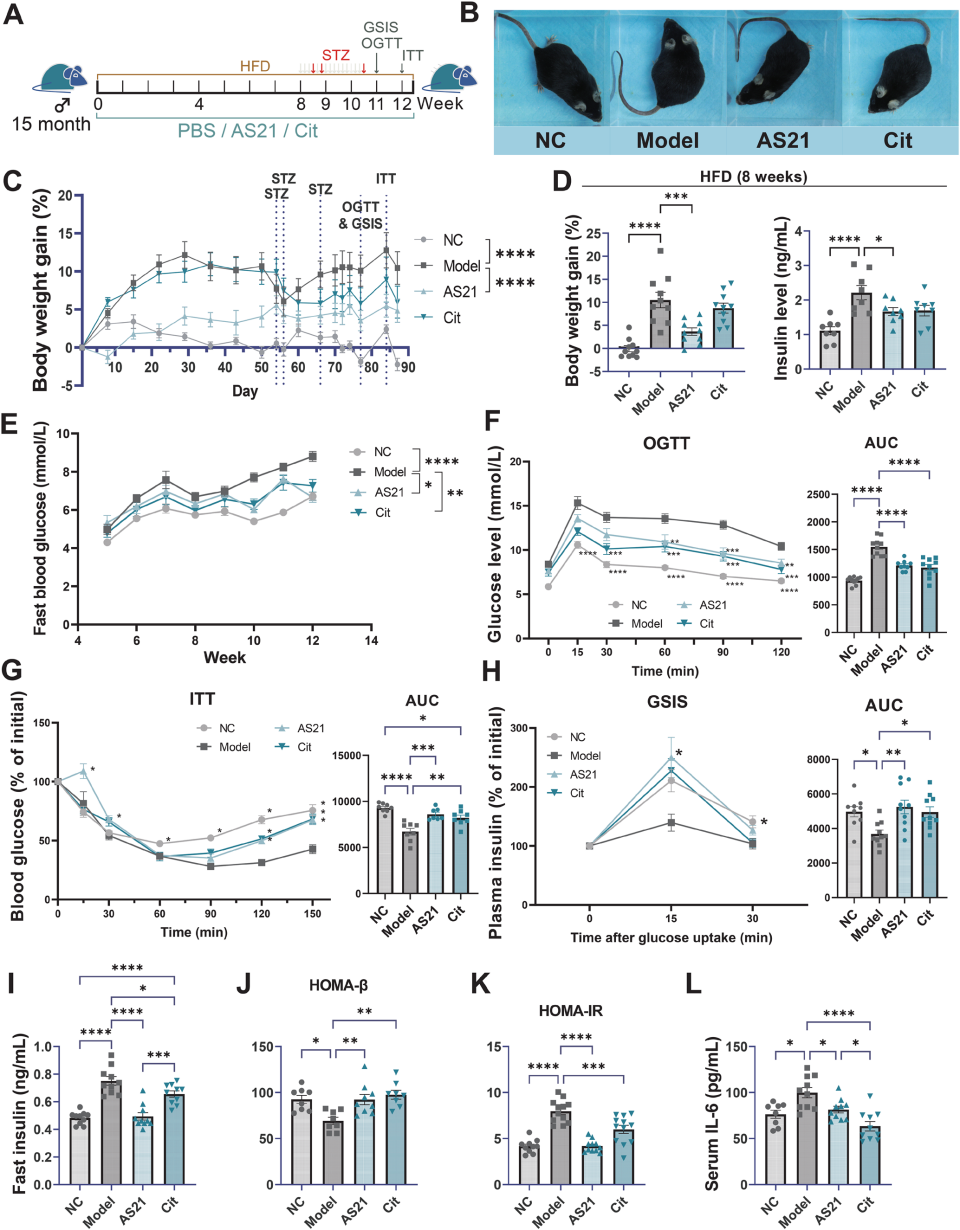
AS21 and citrulline supplementation improve glucose tolerance and insulin secretion in a prediabetic mouse model. (A) Experimental design of HFD/STZ‐induced prediabetic mouse model supplemented with either AS21 or citrulline. (B) Representative images of mice after 8 weeks of HFD feeding. (C) Body weight progression throughout the experiment period. (D) Body weight gain and plasma insulin levels following 8 weeks of HFD induction. (E) Fasting blood glucose levels from Week 5 onward. (F–K) Metabolic assessments at the study endpoint, including the oral glucose tolerance test (OGTT), intraperitoneal insulin tolerance test (ITT), glucose‐stimulated insulin secretion (GSIS), fasting insulin, HOMA‐β, and HOMA‐IR. (L) Serum IL‐6 levels at study endpoint. Statistics: Four‐group comparisons were analyzed using one‐way ANOVA followed by Tukey's post hoc test. *p < 0.05; **p < 0.01; ***p < 0.001; ****p < 0.0001. Data points represent individual biological replicates. AS21, *Lactiplantibacillus plantarum* AS21; AUC, area under the curve; Cit, citrulline; HFD, high‐fat diet; HOMA‐β, homeostatic model assessment of β‐cell function; HOMA‐IR, homeostatic model assessment of insulin resistance; IL‐6, interleukin‐6; PBS, phosphate buffer saline; STZ, streptozotocin.

To further dissect the metabolic impact of citrulline in vivo, we next established a prediabetic mouse model via a high‐fat diet combined with streptozotocin (HFD + STZ) in 15‐month‐old male C57BL/6J mice, using AS21 administration as a reference. While citrulline effectively preserved insulin responsiveness and exerted robust anti‐inflammatory effects, its mitigation of body weight gain was limited. Three weeks of HFD increased body weight by approximately 10%; notably, AS21, but not citrulline, attenuated this initial gain (Figure 5C). Consistently, AS21 protected against long‐term HFD‐induced metabolic disturbances, particularly modulating body weight and plasma insulin after 8 weeks (Figure 5B,D).

Importantly, both AS21 and citrulline treatments blunted the development of impaired fasting glucose (Figure 5E) and significantly improved glucose tolerance and insulin sensitivity, as assessed by OGTT and ITT (Figure 5F,G). During the ITT, blood glucose in control mice rebounded at 30 min, whereas in AS21‐ and citrulline‐treated groups, it rebounded at 60 min and fully recovered to baseline by 150 min. Conversely, model mice exhibited a continuous decline until 90 min, resulting in a prolonged period of hypoglycemia that underscores defective counter‐regulation and impaired islet function (Figure 5G). Notably, citrulline significantly restored GSIS, which was severely compromised in the model group (Figure 5H and Figure S12C), achieving an improvement comparable to that of the AS21. This restoration of GSIS largely accounts for the mitigation of glucose intolerance. Taken together, these data indicate that exogenous citrulline preserves insulin responsiveness and glucose homeostasis despite limited impacts on body weight, whereas AS21 more broadly prevents the overall prediabetic phenotype.

Given the well‐documented anti‐inflammatory properties of citrulline, we validated these systemic effects in our prediabetes model. Both citrulline and AS21 significantly decreased the serum IL‐1β, TNF‐α, and IL‐6 (Figure S12A and Figure 5L). Furthermore, hepatic function indicators were also protected (Figure S12D), whereas renal protective benefits remained limited (Figure S12E).

### 3.7 Citrulline Enhances GLP‐1 Production and Cellular Resilience in Enteroendocrine Cells

**FIGURE 6:**
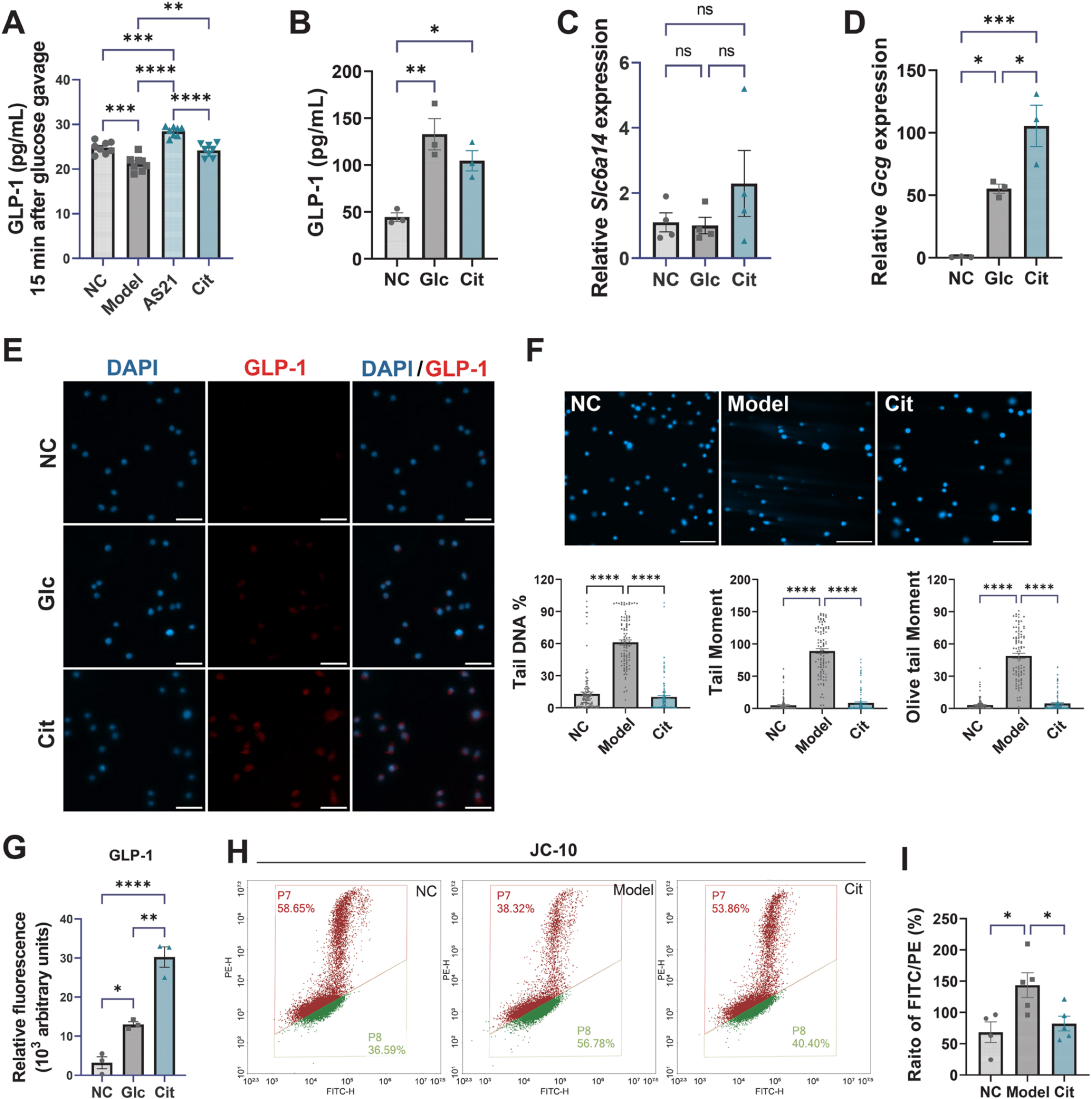
Citrulline promotes GLP‐1 secretion and supports cellular integrity in STC‐1 enteroendocrine cells. (A) Plasma GLP‐1 levels in prediabetic mice. (B) GLP‐1 concentrations in the supernatants of citrulline‐treated STC‐1 cells. (C, D) Relative mRNA expression of *Slc6a14* and *Gcg*. (E) Representative immunofluorescence images of GLP‐1 in STC‐1 cells. Scale bar, 50 μm. (F) Representative comet assay images and quantification of DNA damage. Scale bar, 200 μm. (G) Quantification of GLP‐1 immunofluorescence intensity. (H) Flow cytometry analysis of JC‐10 staining in STC‐1 cell. (I) The FITC/PE fluorescence intensity ratio derived from panel (H), reflecting mitochondrial membrane potential. Statistics: Comparisons were analyzed using one‐way ANOVA with Tukey's post hoc tests. ns, *p* > 0.05; *p < 0.05; **p < 0.01; ***p < 0.001; ****p < 0.0001. Data points represent individual biological replicates. Gcg, proglucagon; Glc, glucose; GLP‐1, glucagon‐like peptide‐1.

To investigate the molecular role of citrulline in modulating GLP‐1 secretion, we utilized the STC‐1 cells as an in vitro enteroendocrine model. Citrulline treatment significantly elevated both active GLP‐1 secretion into the culture supernatant and its intracellular biosynthesis, as evidenced by upregulated *Gcg* expression and heightened immunofluorescence intensity (Figure 6B–E). These cellular results corroborate the in vivo GLP‐1 activation observed in the prediabetic male mice. Furthermore, citrulline is putatively transported by Slc6a14, and we found that citrulline (500 μM) stimulated intracellular GLP‐1 production more robustly than glucose (10 mM), while eliciting comparable secretion outputs.

To assess whether citrulline preserves cellular resilience under detrimental conditions, we subjected STC‐1 cells to UVC irradiation and H_2_O_2_‐induced oxidative stress. Comet assay analysis revealed that citrulline partially mitigated DNA damage, as indicated by significant reductions in tail DNA percentage, tail moment, and Olive tail moment (Figure 6F). Furthermore, JC‐10 staining demonstrated that citrulline alleviated H_2_O_2_‐induced mitochondrial depolarization by approximately 10% (Figure 6H,I). Collectively, these data indicate that, alongside its primary role in stimulating GLP‐1 biosynthesis and secretion, citrulline may help mitigate cellular and mitochondrial stress, thereby preserving enteroendocrine function integrity under pathological stress.

## 4 Discussion

Sex differences represent a fundamental feature of aging biology and contribute to divergent trajectories of metabolic diseases in later life (Hägg and Jylhävä 2021; Yan et al. 2022). However, the biological mechanisms underlying sex‐biased metabolic vulnerability remain poorly understood. Emerging evidence indicates that the gut microbiota plays an important role in shaping host metabolic homeostasis, partly through the regulation of amino acid metabolism (Dwibedi et al. 2025; Jojima et al. 2024; Li et al. 2024). Whether microbiota‐dependent amino acid metabolism contributes to sex‐specific metabolic aging has remained largely unexplored. In this study, we identify a previously unrecognized link between microbial citrulline metabolism and sex‐biased glucose dysregulation during aging. Using naturally aged mice, we show that male animals develop impaired glucose homeostasis accompanied by depletion of circulating citrulline. Probiotic intervention with AS21 partially restores metabolic balance in aged males, an effect associated with enhanced microbial citrulline biosynthetic capacity and increased circulating citrulline levels.

Crucially, we observed distinct baseline aging trajectories between male and female mice, which may account for the sexually dimorphic responses to AS21 intervention. At the host physiological level, aged male mice exhibited accumulated hepatic oxidative stress (Figure S5D) alongside aberrant elevations in ileal *Otc* and renal *Ass1* expression (Figure 2G), both of which corresponded to systemic citrulline depletion. In parallel, the male microbiota showed more pronounced age‐associated shifts in composition (e.g., Campylobacterota and *Helicobacter*) and broader functional alterations, particularly in amino acid metabolism pathways, whereas the female microbial profile remained relatively stable. Together, these findings suggest that sex‐specific differences in host and microbial baseline states shape microbiota‐dependent amino acid metabolism during aging, providing a modifiable axis for AS21 intervention.

An important question is whether age‐associated citrulline depletion represents a causal metabolic disturbance or a secondary consequence of aging. Citrulline is a non‐essential amino acid that reflects systemic metabolic status (Bagheripour et al. 2023) and has been reported to decline with age in mice (Xie et al. 2025). Furthermore, altered citrulline levels are common in metabolic disorders with impaired glucose regulation (Cao et al. 2025; Ding et al. 2021), and supplementation improves glycemic control in various models (Abdelrahman et al. 2024; Capel et al. 2015). However, the interpretation of glucose homeostasis based solely on serum citrulline is potentially confounded by renal function (Ikeda 2022). In our study, the metabolic improvement induced by AS21 in naturally aged and prediabetic mice occurred concurrently with the restoration of circulating citrulline levels (Figures 1 and 5). In contrast, direct citrulline supplementation prevented the onset of glucose intolerance but did not restore steady‐state serum citrulline concentrations (Figure 5 and Figure S12), which may be attributable to the limited capacity of exogenous supplementation to influence renal clearance or intrinsic host metabolism. These observations suggest that citrulline may function both as a metabolically active regulator and a systemic metabolic indicator, with its normal physiological serum range yet to be established.

Increasing evidence indicates that the gut microbiota contributes to host metabolite pools through gene‐encoded metabolic pathways and specific microbial taxa (Li et al. 2024; Qian et al. 2024). In this study, metagenomic analysis traced citrulline synthesis–related pathways predominantly to mucosa‐associated microbial taxa, including *Helicobacter* and *Veillonella* (Figure 3E,F and Figure S11). Consistently, AS21 administration enriched citrulline biosynthesis–related functional genes in vivo and increased citrulline production in vitro mucosal microbial co‐culture systems devoid of host factors (Figure 4). Although AKG was hypothesized to serve as a precursor, supplementation alone did not fully recapitulate the effects of live AS21, because it did not increase the abundance of citrulline‐producing taxa or their associated functional genes (Figure 5 and Figure S10). Thus, a single precursor supply cannot provide the continuous metabolic or induce sustained ecological shifts necessary for robust microbial citrulline synthesis. This highlights community remodeling—rather than mere substrate provision—as the primary mechanism driving AS21 efficacy. Notably, citrulline production in the cell‐free group further suggests that enzymatically active components may contribute to short‐term synthesis (Figure 4B). Collectively, these findings demonstrate that AS21 enhances the intrinsic metabolic capacity of the mucosal microbiota to synthesize citrulline. Interestingly, although AS21 induced broadly parallel alterations in amino acid metabolic pathways in both sexes, the enrichment of citrulline synthesis genes reached statistical significance only in males (Figure 3, Figures S7 and S8). This pattern suggests that sex differences reflect baseline disparities in metabolic and microbial states rather than opposing regulatory responses to the probiotic. Microbiota‐dependent metabolic plasticity may thus buffer sex‐specific metabolic vulnerabilities during aging (Zhang et al. 2021).

The restoration of citrulline metabolism was associated with several beneficial downstream metabolic effects. In prediabetic mice, citrulline supplementation reduced systemic inflammatory markers and improved glucose tolerance, accompanied by enhanced GSIS (Figure 5 and Figure S12). Systemic GLP‐1 levels were also elevated (Figure 6A). Previous studies examining the physiological functions of citrulline have predominantly focused on its anti‐inflammatory properties (Mao et al. 2022; Xie et al. 2025), pancreatic β‐cell activity, and mitochondrial metabolism (Bagheripour et al. 2023; Papadia et al. 2018). However, gut‐derived metabolites are increasingly recognized as regulators of enteroendocrine signaling. For example, short‐chain fatty acids (Shaheen et al. 2025; Yao et al. 2020) and bile acids (Yao et al. 2020) stimulate GLP‐1 secretion through defined receptor‐mediated pathways. In STC‐1 cells, citrulline stimulated GLP‐1 secretion, potentially via the amino acid transporter Slc6a14 (Figure 6), suggesting a link between microbial amino acid metabolism and incretin regulation.

Translational evidence from clinical cohorts remains limited (Figure S13). Despite the paucity of studies, available reports generally point toward the metabolic benefits of exogenous citrulline supplementation (Table S1). However, high‐dose administration of free citrulline has occasionally been associated with gastrointestinal discomfort (Holguin et al. 2019). In contrast, dietary sources, such as watermelon, appear to facilitate a more favorable, physiological exposure (Shanely et al. 2020; Vincellette et al. 2021). These findings suggest that maintaining citrulline within a physiological range may be critical for maximizing its metabolic benefit. Microbiota‐targeted strategies that modulate endogenous citrulline metabolism may therefore represent a more physiologically relevant approach. Consistently, aged females with higher baseline citrulline levels did not respond further to AS21, whereas aged males with depletion showed restored microbial synthesis capacity and improved metabolic outcomes (Figures 2 and 3). Together, these findings suggest that AS21‐mediated modulation of citrulline metabolism represents a physiologically balanced and context‐dependent strategy.

Importantly, our findings propose a mechanistic axis linking AS21 intervention to host metabolic improvements via the restoration of mucosa‐associated citrulline production. First of all, the parallel rescues of circulating citrulline and glucose tolerance were observed in aged males administered AS21. Additionally, AS21‐modulated mucosal microbiome enhanced in vitro citrulline synthesis capacity. Moreover, both AS21 and citrulline supplementation achieved a similar effect on maintaining glucose homeostasis in male‐prediabetic models (e.g., improved fasting glucose, glucose tolerance, and GSIS). However, several limitations of the current study warrant consideration. Critically, definitive in vivo causal validation is currently lacking. For instance, genetic ablation of mucosal microbial citrulline biosynthesis pathways or pharmacological disruption of host citrulline utilization. Additionally, in vivo metabolic flux analysis is needed to define host versus microbiota contributions to the circulating citrulline pool. It also remains to be fully elucidated whether the observed sex‐biased response to AS21 is driven primarily by baseline disparities in the initial gut microbiota landscape. Lastly, large‐scale human cohort analyses and clinical intervention trials are required to assess translational relevance. Future studies integrating longitudinal human metabolic profiling will be necessary to clarify the age‐dependent trajectory of circulating citrulline and defining its relationship with glucose homeostasis.

In summary, our study reveals sex‐specific alterations in circulating citrulline during aging, with males showing depletion associated with impaired glucose homeostasis. Probiotic AS21 ameliorates glucose dysregulation through microbiota remodeling and enhanced microbial citrulline biosynthetic capacity in aged males. Complementary evidence from supplementation and cellular models further supports a potential role for citrulline in incretin‐mediated glucose regulation. Collectively, these findings highlight the significance of microbiota‐associated amino acid metabolism in the context of sex‐biased metabolic aging, positioning AS21 as a potential microbiota‐targeted strategy for metabolic improvement.

## Author Contributions

M.J., M.C., and H.C. designed and performed the experiments, analyzed the data, and drafted the manuscript. H.Z., R.D., and C.W. performed the experiments and contributed to methodology development. M.I. and X.W. provided resources and methodological support. Z.D. conceptualized the study, supervised the project, and managed project administration. X.G. conceptualized the study, acquired funding, supervised the project, and revised the manuscript. All authors reviewed and approved the final manuscript.

## Funding

This work was supported by the Major Science and Technology Project of Gansu Province (23ZDKA0007).

## Conflicts of Interest

The authors declare no conflicts of interest.

## Supporting information


**Figure S1:** Extended metabolic characterization of naturally aged mice treated with AS21. (A, B) Body weight progression during the intervention period in male (A) and female (B) mice. (C) Food intake progression during the intervention period. (D) Average water intake during the final week of the intervention. (E) Serum leptin (LEP) levels. (F) Water intake progression during the intervention period. (G) HOMA‐β index. (H) Serum IL‐1β levels. (I) Intraperitoneal insulin tolerance tests (ITT) in male and female mice. (J) Area under the curve (AUC) for the ITT. Statistics: Two‐way ANOVA was performed with sex and experimental group (20m, 24m, and 24m_AS21) as independent factors unless otherwise specified. Longitudinal data are presented as time‐course curves. Area under the curve (AUC) values for body weight, food intake and water intake were calculated and compared using one‐way ANOVA within each sex. For ITT analyses, data from male and female mice were analyzed separately at each time point using ordinary one‐way ANOVA or the Kruskal–Wallis test, as dictated by data normality. Overall *p*‐value summaries for each time point are presented in (I). ns, *p* > 0.05; *p < 0.05; **p < 0.01. Data points represent individual biological replicates. 6m_OGTT, oral glucose tolerance test after 6‐month intervention; 6m_ITT, insulin tolerance test after 6‐month intervention; HOMA‐β, homeostatic model assessment of β‐cell function; IL‐1β, interleukin‐1β.

**Figure S2:** Detailed multiple‐comparison results of the OGTT in naturally aged mouse model. Detailed multiple comparisons of each experimental group against the corresponding 24‐month‐old (24 m) control group in male (A) and female (B) mice. ns, *p* > 0.05; *p < 0.05; **p < 0.01; ***p < 0.001; ****p < 0.0001. Data points represent individual biological replicates. OGTT, oral glucose tolerance test.

**Figure S3:** Detailed multiple‐comparison results of ITT in the naturally aged mouse model. Detailed multiple comparisons of each experimental group against the corresponding 24‐month‐old (24 m) control group in male (A) and female (B) mice. ns, *p* > 0.05; *p < 0.05. Data points represent individual biological replicates. ITT, insulin tolerance test.

**Figure S4:** Heatmap of differentially abundant metabolites and KEGG over‐representation analysis (ORA). (A) Heatmap showing the relative abundance (z‐score normalized) of sexually dimorphic metabolites in 24‐month‐old mice. (B) Pathway enrichment results for aging‐, sex‐ and AS21 intervention‐associated differentially abundant metabolites.

**Figure S5:** Extended biochemical characterization of naturally aging mice. (A) Schematic diagram detailing sample collection of naturally aged mice at the indicated ages. (B) Serum citrulline concentrations quantified via ELISA. (C) Spearman correlation analysis between br08001‐annotated metabolites (see Figure 2A) and measured metabolic parameters. (D) Hepatic superoxide dismutase (SOD) activity and malondialdehyde (MDA) levels. (E) Serum aspartate aminotransferase (AST), total bilirubin (TB) and blood urea nitrogen (BUN) levels. (F) Citrulline content in ileal and colonic tissues quantified via ELISA. (G) Hepatic *Asl* mRNA expression. (H) Renal *Ass1* mRNA expression. Statistics: Data were analyzed using two‐way ANOVA followed by Tukey's post hoc test unless otherwise specified. Correlation analysis were performed utilizing Spearman's rank correlation coefficient. ns, *p* > 0.05; *p < 0.05; **p < 0.01; ***p < 0.001; ****p < 0.0001. Data points represent individual biological replicates. APN, Adiponectin; IFN‐γ, interferon‐γ; IL‐1β, interleukin‐1β; IL‐10, interleukin‐10; IL‐6, interleukin‐6; ITT AUC, area under the curve for the intraperitoneal insulin tolerance test; LEP, Leptin; OGTT AUC, area under the curve for the oral glucose tolerance test; TNF‐α, tumor necrosis factor‐α.

**Figure S6:** Gut mucosal microbiota structure in naturally aged mice following AS21 intervention. (A) α‐diversity analysis of the gut mucosal microbiota in male and female mice. (B) β‐diversity analysis based on Bray–Curtis distance in male and female mice. (C, D) Relative abundance of the gut mucosal microbiota at the indicated taxonomic levels. (E) UpSet plot analysis showing shared and unique taxa among the experimental groups. Statistics: α‐diversity indices were compared between sexes within each experimental group using the Mann–Whitney *U* test. β‐diversity differences between sexes within each experimental group were assessed utilizing PERMANOVA based on Bray‐Curtis distances. ns, *p* > 0.05; *p < 0.05; **p < 0.01. Data points represent individual biological replicates.

**Figure S7:** Sex‐ and age‐associated metagenomic features of the gut mucosal microbiota in naturally aged mice. (A) Heatmap of differentially abundant mucosal genera identified across sex‐, aging‐, and AS21 intervention‐associated comparisons. Color annotations indicate the specific comparison categories. (B) KEGG pathway enrichment analysis of differentially abundant functional genes within the mucosal microbiota. (C, D) Spearman correlation analysis between differentially abundant mucosal species and serum metabolites in female (C) and male (D) mice. (E) Relative abundance of *Helicobacter typhlonius* . Statistics: Differential abundant taxa were identified using appropriate non‐parametric tests with multiple‐comparison correction where applicable. Panel (E) was analyzed utilizing the Mann–Whitney *U* test for pairwise comparisons. Correlation analyses were performed using Spearman's rank correlation coefficient. ns, *p* > 0.05; *p < 0.05; **p < 0.01. Data points represent individual biological replicates. In panel (B): 20(FvsM), 20‐month‐old females versus 20‐month‐old males; 24(FvsM), 24‐month‐old females versus 24‐month‐old males; 24P(FvsM), 24‐month‐old females with AS21 versus 24‐month‐old males with AS21; F(20vs24), 20‐month‐old versus 24‐month‐old females; M(20vs24), 20‐month‐old versus 24‐month‐old males; F(24vs24P), 24‐month‐old females versus 24‐month‐old females with AS21; M(24vs24P), 24‐month‐old males versus 24‐month‐old males with AS21.

**Figure S8:** Relative abundance of KEGG orthologs involved in microbial citrulline metabolism within gut mucosal microbiota. (A) Relative abundance of eight KEGG orthologs associated with microbial citrulline metabolic pathways in 24‐month‐old female and male mice, as well as their corresponding AS21‐treated groups. Statistics: Pre‐specified pairwise comparisons were conducted using the two‐tailed Mann–Whitney *U* test for the following contrasts: 24‐month‐old females versus 24‐month‐old males; 24‐month‐old females versus AS21‐treated females; and 24‐month‐old males versus AS21‐treated males. ns, *p* > 0.05; *p < 0.05; **p < 0.01. Data points represent individual biological replicates.

**Figure S9:** Metagenome‐assembled genomes (MAGs) of *Helicobacter apodemus*. (A) Circular genomic map of MAG.H58.6, assigned to *Helicobacter apodemus*. (B) Stacked bar chart showing the number of KEGG‐annotated genes involved in citrulline synthesis. (C) Phylogenetic tree based on the BLASTp of the ornithine carbamoyltransferase sequence derived from MAG.H58.6.

**Figure S10:** Functional validation of microbial citrulline metabolism in ex vivo co‐culture systems. (A) Heatmap of differentially abundant phyla and species across co‐cultured treatments. (B) Relative abundance of KEGG orthologs involved in citrulline biosynthesis within the co‐cultured microbiota. (C) Citrulline concentrations in culture supernatants following 24 h of cultivation. (D) Glutamate and glutamine concentrations in culture supernatants at indicated time points. (E) α‐ketoglutarate (AKG) abundance in culture supernatants at 24 and 48 h in AKG‐supplemented cultures. (F) Relative abundance of KEGG orthologs involved in citrulline biosynthesis in AKG‐supplemented groups. (G) Relative abundance of metabolites associated with citrulline biosynthesis in the AS21 culture supernatant. Statistics: Paired two‐tailed *t*‐tests were utilized for comparisons between 24 and 48 h within the same culture condition. Unpaired two‐tailed *t*‐tests were used for two‐group comparisons. Multiple‐group comparisons were analyzed via ordinary one‐way ANOVA followed by Dunnett's post hoc test against the designated control group. Statistical significance is indicated in the figures. In panel (B): *p < 0.05 and **p < 0.01 versus the MRS group; ^#^ *p* < 0.05 and ^##^ *p* < 0.01 versus the AS21 group. Data points represent individual biological replicates. A_cf, cell‐free lysate of AS21; A_sn, AS21 culture supernatant; A_hk, heat‐killed AS21; AS21, live AS21 strain; AKG, α‐ketoglutarate; MRS, de Man, Rogosa and Sharpe medium.

**Figure S11:** Species‐level source tracking of citrulline biosynthesis genes in the in vitro co‐culture systems. (A) Species‐level source tracking of citrulline biosynthesis‐related KEGG orthologs in MRS and A_cf groups. (B) Species‐level source tracking of citrulline biosynthesis‐related KEGG orthologs in the AS21, A_sn and A_hk groups. Abbreviations: MRS, de Man, Rogosa and Sharpe medium; A_cf, cell‐free lysate of AS21; A_sn, AS21 culture supernatant; A_hk, heat‐killed AS21; AS21, live AS21 strain.

**Figure S12:** Metabolic assessment of prediabetic model mice. (A) Serum inflammatory cytokines, including IL‐1β, TNF‐α, and IL‐6. (B) Serum citrulline levels. (C) Plasma insulin levels following oral glucose gavage. (D) Serum aspartate aminotransferase (AST), and alanine aminotransferase (ALT) levels. (E) Serum blood urea nitrogen (BUN) levels. Statistics: Four‐group comparisons were analyzed utilizing one‐way ANOVA followed by Tukey's post hoc test. *p < 0.05; **p < 0.01; ***p < 0.001; ****p < 0.0001. Data points represent individual biological replicates. AS21, *Lactiplantibacillus plantarum* AS21; Cit, citrulline; IL‐1β, interleukin‐1β; TNF‐α, tumor necrosis factor‐α.

**Figure S13:** Literature screening workflow following PRISMA guidelines. Flowchart illustrating the identification, screening, eligibility assessment, and inclusion of studies in the systematic literature review.

**Data S1:** Supplementary methods.

**Table S1:** Summary of clinical studies investigating citrulline supplementation and glycemic outcomes.

**Table S2:** Primer sequences for the qPCR.

**Table S3:** Protein sequence for ArgF used in Phylogenetic tree.

**Data S2:** Aging cell author checklist.

## Data Availability

Metagenomic sequencing data for mucosal microbiota and in vitro co‐culturation from this study have been deposited at the NCBI Sequence Read Archive (https://www.ncbi.nlm.nih.gov) under Bioproject PRJNA1417735 and PRJNA1418210. Raw data for serum metabolomics have been deposited at Metabolomics Workbench (https://www.metabolomicsworkbench.org) under ST004699. The assembled genome sequence has been deposited at GenBank (https://www.ncbi.nlm.nih.gov/genbank) under accession number JCACOA000000000. Any other data reported in this work are available from the corresponding author upon reasonable request.

## References

[acel70662-bib-0002] Abdelrahman, A. A. , P. Sandow , J. Wang , et al. 2024. “Arginine Deprivation/Citrulline Augmentation With ADI‐PEG20 as Novel Therapy for Complications in Type 2 Diabetes.” Molecular Metabolism 89: 102020. 10.1016/j.molmet.2024.102020. 39214514 PMC11414555

[acel70662-bib-0005] Bagheripour, F. , S. Jeddi , K. Kashfi , and A. Ghasemi . 2023. “Metabolic Effects of L‐Citrulline in Type 2 Diabetes.” Acta Physiologica 237, no. 3: e13937. 10.1111/apha.13937. 36645144

[acel70662-bib-0006] Bauer, P. V. , F. A. Duca , T. M. Z. Waise , et al. 2018. “ *Lactobacillus gasseri* in the Upper Small Intestine Impacts an ACSL3‐Dependent Fatty Acid‐Sensing Pathway Regulating Whole‐Body Glucose Homeostasis.” Cell Metabolism 27, no. 3: 572–587. 10.1016/j.cmet.2018.01.013. 29514066

[acel70662-bib-0007] Birkenfeld, A. L. , and V. Mohan . 2024. “Prediabetes Remission for Type 2 Diabetes Mellitus Prevention.” Nature Reviews Endocrinology 20, no. 8: 441–442. 10.1038/s41574-024-00996-8. 38806698

[acel70662-bib-0008] Cao, B. , H. Lu , P. Liu , Y. Zhang , and C. Wang . 2025. “Serum Metabolomics Signature of Maternally Inherited Diabetes and Deafness by Gas Chromatography‐Time of Flight Mass Spectrometry.” Journal of Diabetes Investigation 16, no. 1: 146–153. 10.1111/jdi.14334. 39480690 PMC11693542

[acel70662-bib-0009] Capel, F. , G. Chabrier , E. Pitois , et al. 2015. “Combining Citrulline With Atorvastatin Preserves Glucose Homeostasis in a Murine Model of Diet‐Induced Obesity.” British Journal of Pharmacology 172, no. 20: 4996–5008. 10.1111/bph.13269. 26228176 PMC4621993

[acel70662-bib-0010] Chakkalakal, R. J. , K. I. Galaviz , S. Thirunavukkarasu , M. K. Shah , and K. M. V. Narayan . 2024. “Test and Treat for Prediabetes: A Review of the Health Effects of Prediabetes and the Role of Screening and Prevention.” Annual Review of Public Health 45: 151–167. 10.1146/annurev-publhealth-060222-023417. 38109519

[acel70662-bib-0011] Ding, Y. , M. C. Haks , G. Forn‐Cuní , et al. 2021. “Metabolomic and Transcriptomic Profiling of Adult Mice and Larval Zebrafish Leptin Mutants Reveal a Common Pattern of Changes in Metabolites and Signaling Pathways.” Cell & Bioscience 11, no. 1: 126. 10.1186/s13578-021-00642-0. 34233759 PMC8265131

[acel70662-bib-0012] Dwibedi, C. , A. S. Axelsson , B. Abrahamsson , et al. 2025. “Effect of Broccoli Sprout Extract and Baseline Gut Microbiota on Fasting Blood Glucose in Prediabetes: A Randomized, Placebo‐Controlled Trial.” Nature Microbiology 10, no. 3: 681–693. 10.1038/s41564-025-01932-w. PMC11879859 39929977

[acel70662-bib-0013] Hägg, S. , and J. Jylhävä . 2021. “Sex Differences in Biological Aging With a Focus on Human Studies.” eLife 10: e63425. 10.7554/eLife.63425. 33982659 PMC8118651

[acel70662-bib-0014] Holguin, F. , H. Grasemann , S. Sharma , et al. 2019. “L‐Citrulline Increases Nitric Oxide and Improves Control in Obese Asthmatics.” JCI Insight 4, no. 24: e131733. 10.1172/jci.insight.131733. 31714895 PMC6975256

[acel70662-bib-0015] Huang, Y. , H. Lu , L. Zhang , et al. 2025. “Dietary Selenium Deficiency Accelerates the Onset of Aging‐Related Gut Microbial Changes in Aged Telomere‐Humanized Mice, With *Akkermansia muciniphila* Being the Most Prominent and Alleviating Selenium Deficiency‐Induced Type 2 Diabetes.” Aging Cell 24, no. 8: e70130. 10.1111/acel.70130. 40540389 PMC12341817

[acel70662-bib-0016] Ikeda, H. 2022. “The Effect of Mild Renal Dysfunction on the Assessment of Plasma Amino Acid Concentration and Insulin Resistance in Patients With Type 2 Diabetes Mellitus.” Journal of Diabetes Research 2022: 2048300. 10.1155/2022/2048300. 35734236 PMC9208954

[acel70662-bib-0017] International Diabetes Federation . 2025. “IDF Diabetes Atlas 11th Edition.” https://diabetesatlas.org/resources/idf‐diabetes‐atlas‐2025.

[acel70662-bib-0018] Ji, H. , A. C. Kwan , M. Chen , et al. 2022. “Sex Differences in Myocardial and Vascular Aging.” Circulation Research 130, no. 4: 566–577. 10.1161/CIRCRESAHA.121.319902. 35175845 PMC8863105

[acel70662-bib-0019] Jojima, T. , S. Sakurai , H. Kishi , et al. 2024. “Empagliflozin Increases Plasma Levels of Citrulline, Histidine, and α‐Aminobutyric Acid in Patients With Type 2 Diabetes: Effects of a Sodium‐Glucose Co‐Transporter 2 Inhibitor on the Plasma Amino Acid Profile.” Expert Opinion on Pharmacotherapy 25, no. 7: 937–944. 10.1080/14656566.2024.2362265. 38809611

[acel70662-bib-0021] Kroemer, G. , A. B. Maier , A. M. Cuervo , et al. 2025. “From Geroscience to Precision Geromedicine: Understanding and Managing Aging.” Cell 188, no. 8: 2043–2062. 10.1016/j.cell.2025.03.011. 40250404 PMC12037106

[acel70662-bib-0022] Li, T. , X. Chen , D. Huo , et al. 2024. “Microbiota Metabolism of Intestinal Amino Acids Impacts Host Nutrient Homeostasis and Physiology.” Cell Host & Microbe 32, no. 5: 661–675. 10.1016/j.chom.2024.04.004. 38657606 PMC11636940

[acel70662-bib-0023] Li, W. , L. Gao , W. Huang , et al. 2022. “Antioxidant Properties of Lactic Acid Bacteria Isolated From Traditional Fermented Yak Milk and Their Probiotic Effects on the Oxidative Senescence of *Caenorhabditis elegans* .” Food & Function 13, no. 6: 3690–3703. 10.1039/d1fo03538j. 35262535

[acel70662-bib-0024] Mao, Y. , D. Shi , G. Li , and P. Jiang . 2022. “Citrulline Depletion by ASS1 Is Required for Proinflammatory Macrophage Activation and Immune Responses.” Molecular Cell 82, no. 3: 527–541. 10.1016/j.molcel.2021.12.006. 35016033

[acel70662-bib-0025] Page, M. J. , J. E. McKenzie , P. M. Bossuyt , et al. 2021. “The PRISMA 2020 Statement: An Updated Guideline for Reporting Systematic Reviews.” BMJ 372: n71. 10.1136/bmj.n71. 33782057 PMC8005924

[acel70662-bib-0026] Papadia, C. , S. Osowska , L. Cynober , and A. Forbes . 2018. “Citrulline in Health and Disease. Review on Human Studies.” Clinical Nutrition 37, no. 6: 1823–1828. 10.1016/j.clnu.2017.10.009. 29107336

[acel70662-bib-0027] Qian, X. , Q. Li , H. Zhu , et al. 2024. “Bifidobacteria With Indole‐3‐Lactic Acid‐Producing Capacity Exhibit Psychobiotic Potential via Reducing Neuroinflammation.” Cell Reports Medicine 5: 101798. 10.1016/j.xcrm.2024.101798. 39471819 PMC11604549

[acel70662-bib-0028] Qin, W. , S. Zheng , L. Zhou , et al. 2025. “High‐Coverage Metabolomics Reveals Gut Microbiota‐Related Metabolic Traits of Type‐2 Diabetes in Serum.” Journal of Proteome Research 24, no. 4: 1649–1661. 10.1021/acs.jproteome.4c00507. 40130449

[acel70662-bib-0029] Sargin, P. , M. F. Roethle , S. Jia , et al. 2022. “ *Lactiplantibacillus plantarum* 299v Supplementation Modulates β‐Cell ER Stress and Antioxidative Defense Pathways and Prevents Type 1 Diabetes in Gluten‐Free BioBreeding Rats.” Gut Microbes 14, no. 1: 2136467. 10.1080/19490976.2022.2136467. 36261888 PMC9586621

[acel70662-bib-0030] Shaheen, N. , W. Khursheed , B. Gurung , and S. Wang . 2025. “ *Akkermansia muciniphila* : A Key Player in Gut Microbiota‐Based Disease Modulation.” Microbiological Research 301: 128317. 10.1016/j.micres.2025.128317. 40845731

[acel70662-bib-0031] Shanely, R. A. , J. J. Zwetsloot , T. J. Jurrissen , et al. 2020. “Daily Watermelon Consumption Decreases Plasma sVCAM‐1 Levels in Overweight and Obese Postmenopausal Women.” Nutrition Research 76: 9–19. 10.1016/j.nutres.2020.02.005. 32142970

[acel70662-bib-0033] Soleimani, A. , M. Zarrati Mojarrad , F. Bahmani , et al. 2017. “Probiotic Supplementation in Diabetic Hemodialysis Patients Has Beneficial Metabolic Effects.” Kidney International 91, no. 2: 435–442. 10.1016/j.kint.2016.09.040. 27927601

[acel70662-bib-0034] Song, C. , Z. Chai , S. Chen , H. Zhang , X. Zhang , and Y. Zhou . 2023. “Intestinal Mucus Components and Secretion Mechanisms: What We Do and Do Not Know.” Experimental and Molecular Medicine 55, no. 4: 681–691. 10.1038/s12276-023-00960-y. 37009791 PMC10167328

[acel70662-bib-0035] Sun, L. , Z. Li , C. Hu , et al. 2023. “Age‐Dependent Changes in the Gut Microbiota and Serum Metabolome Correlate With Renal Function and Human Aging.” Aging Cell 22, no. 12: e14028. 10.1111/acel.14028. 38015106 PMC10726799

[acel70662-bib-0036] Vincellette, C. M. , J. Losso , K. Early , G. Spielmann , B. A. Irving , and T. D. Allerton . 2021. “Supplemental Watermelon Juice Attenuates Acute Hyperglycemia‐Induced Macro‐ and Microvascular Dysfunction in Healthy Adults.” Journal of Nutrition 151, no. 11: 3450–3458. 10.1093/jn/nxab279. 34510203 PMC8562079

[acel70662-bib-0037] Wang, G. , Y. Fan , G. Zhang , et al. 2024. “Microbiota‐Derived Indoles Alleviate Intestinal Inflammation and Modulate Microbiome by Microbial Cross‐Feeding.” Microbiome 12, no. 1: 59. 10.1186/s40168-024-01750-y. 38504383 PMC10949743

[acel70662-bib-0038] Wang, J. , S. Chang , Z. Jin , et al. 2025. “ *Lactobacillus reuteri* ‐Enriched Eicosatrienoic Acid Regulates Glucose Homeostasis by Promoting GLP‐1 Secretion to Protect Intestinal Barrier Integrity.” Journal of Agricultural and Food Chemistry 73, no. 1: 393–408. 10.1021/acs.jafc.4c03818. 39680859

[acel70662-bib-0039] Xia, J. , W. Liu , Y. Ni , et al. 2025. “Advances in the Impact of ASS1 Dysregulation on Metabolic Reprogramming of Tumor Cells.” Cellular Signalling 127: 111593. 10.1016/j.cellsig.2025.111593. 39778698

[acel70662-bib-0040] Xie, Z. , M. Lin , B. Xing , et al. 2025. “Citrulline Regulates Macrophage Metabolism and Inflammation to Counter Aging in Mice.” Science Advances 11, no. 10: eads4957. 10.1126/sciadv.ads4957. 40053596 PMC11887811

[acel70662-bib-0041] Yan, Y. , T. Wu , M. Zhang , C. Li , Q. Liu , and F. Li . 2022. “Prevalence, Awareness and Control of Type 2 Diabetes Mellitus and Risk Factors in Chinese Elderly Population.” BMC Public Health 22, no. 1: 1382. 10.1186/s12889-022-13759-9. 35854279 PMC9295461

[acel70662-bib-0042] Yao, Y. , L. Yan , H. Chen , N. Wu , W. Wang , and D. Wang . 2020. “Cyclocarya Paliurus Polysaccharides Alleviate Type 2 Diabetic Symptoms by Modulating Gut Microbiota and Short‐Chain Fatty Acids.” Phytomedicine 77: 153268. 10.1016/j.phymed.2020.153268. 32663709

[acel70662-bib-0043] Zhang, X. , H. Zhong , Y. Li , et al. 2021. “Sex‐ and Age‐Related Trajectories of the Adult Human Gut Microbiota Shared Across Populations of Different Ethnicities.” Nature Aging 1, no. 1: 87–100. 10.1038/s43587-020-00014-2. 37118004

[acel70662-bib-0044] Zheng, X. , T. Chen , R. Jiang , et al. 2021. “Hyocholic Acid Species Improve Glucose Homeostasis Through a Distinct TGR5 and FXR Signaling Mechanism.” Cell Metabolism 33, no. 4: 791–803. 10.1016/j.cmet.2020.11.017. 33338411

[acel70662-bib-0045] Zhu, T. , and M. O. Goodarzi . 2020. “Metabolites Linking the Gut Microbiome With Risk for Type 2 Diabetes.” Current Nutrition Reports 9, no. 2: 83–93. 10.1007/s13668-020-00307-3. 32157661 PMC7282969

