# Supplementary material for: Lactiplantibacillus plantarum AS21 Attenuates Sex‐Biased Glucose Intolerance in Aging Mice via Gut Mucosal Microbiota‐Derived Citrulline
Source: Aging Cell. 2026 Aug 13;25(8):e70662. doi: 10.1111/acel.70662 (PMC13473181; doi:10.1111/acel.70662)
Supplement: Supplementary file 1 [file ACEL-25-e70662-s001.pdf]

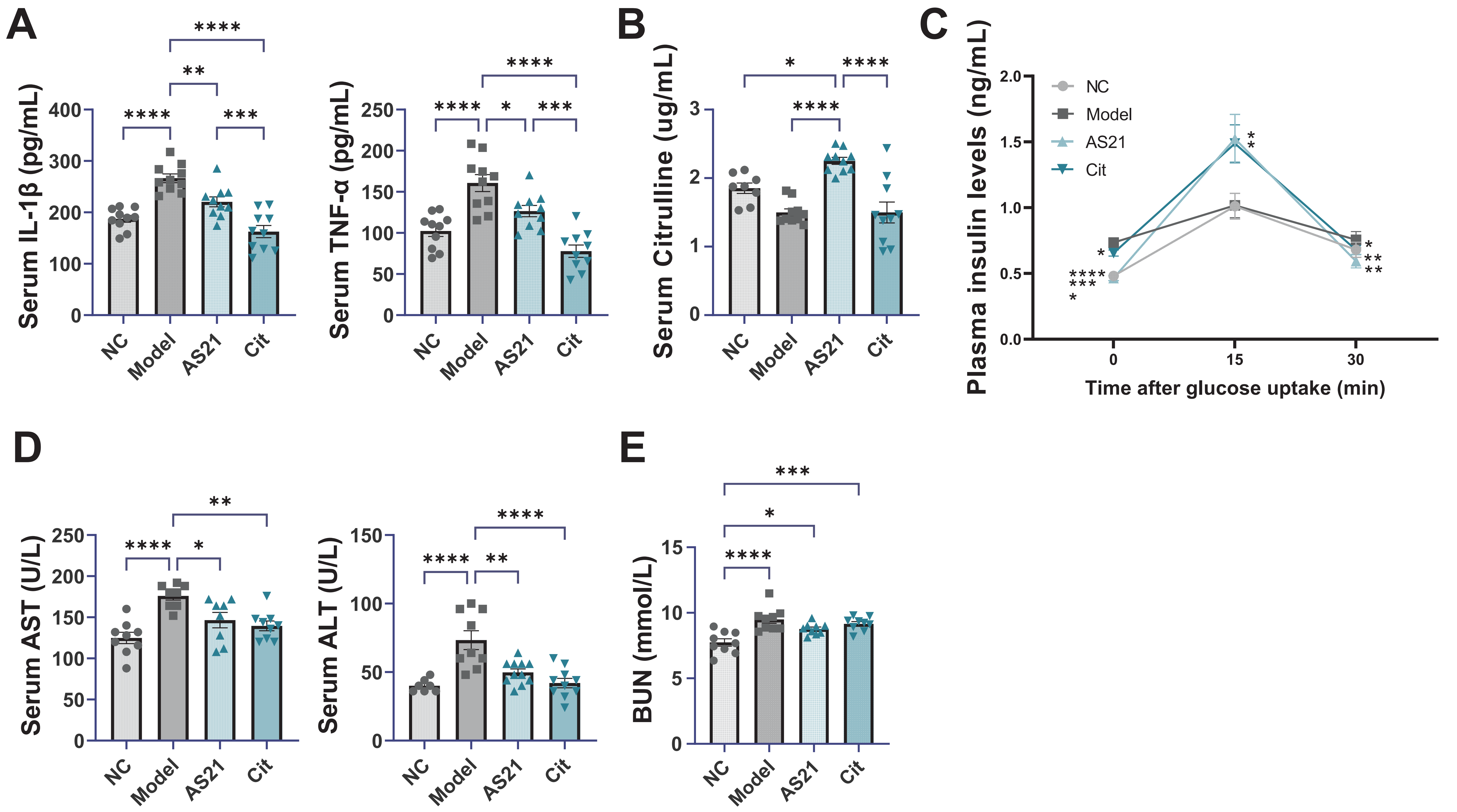

A
Serum IL-1β (pg/mL)
Serum TNF-α (pg/mL)
B
Serum Citrulline (ug/mL)
C
Plasma insulin levels (ng/mL)
NC
Model
AS21
Cit
Time after glucose uptake (min)
D
Serum AST (U/L)
Serum ALT (U/L)
E
BUN (mmol/L)
