# Supplementary material for: Lactiplantibacillus plantarum AS21 Attenuates Sex‐Biased Glucose Intolerance in Aging Mice via Gut Mucosal Microbiota‐Derived Citrulline
Source: Aging Cell. 2026 Aug 13;25(8):e70662. doi: 10.1111/acel.70662 (PMC13473181; doi:10.1111/acel.70662)
Supplement: Supplementary file 2 [file ACEL-25-e70662-s002.pdf]

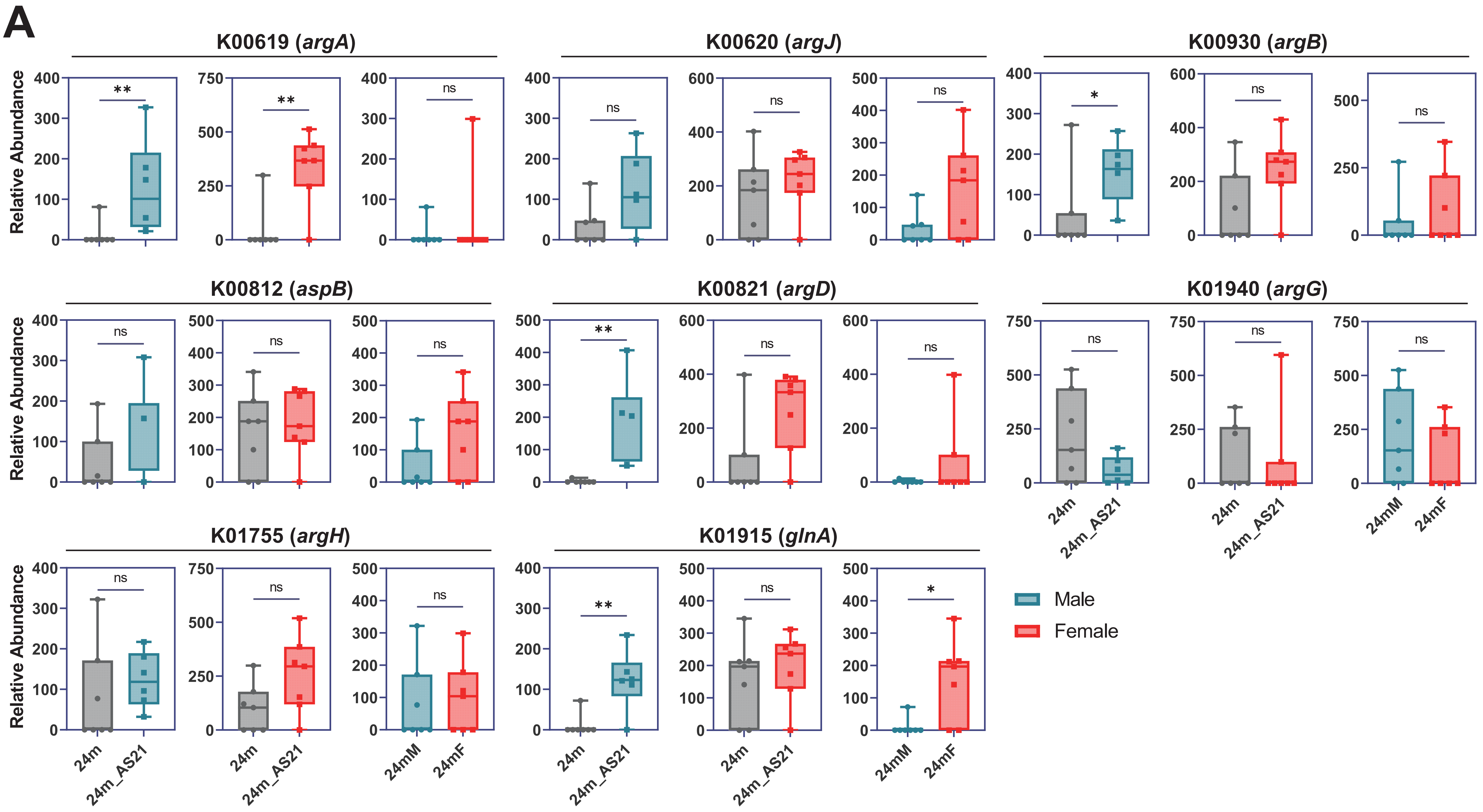
A
K00619 (argA)
K00620 (argJ)
K00930 (argB)
K00812 (aspB)
K00821 (argD)
K01940 (argG)
K01755 (argH)
K01915 (glnA)
Relative Abundance
**
*
ns
24m
24m_AS21
24mM
24mF
Male
Female
