# Supplementary material for: Lactiplantibacillus plantarum AS21 Attenuates Sex‐Biased Glucose Intolerance in Aging Mice via Gut Mucosal Microbiota‐Derived Citrulline
Source: Aging Cell. 2026 Aug 13;25(8):e70662. doi: 10.1111/acel.70662 (PMC13473181; doi:10.1111/acel.70662)
Supplement: Supplementary file 3 [file ACEL-25-e70662-s004.pdf]

AGING CELL AUTHOR CHECKLIST. ***Authors should submit this checklist together with their manuscript. Please ensure that you have read the Author Guidelines in detail before submission.***

**Title**	*Lactiplantibacillus plantarum* AS21 attenuates sex-biased glucose intolerance in aging mice via gut mucosal microbiota-derived citrulline
**Authors**	Mengya Jia, Hu Chen, Mengyan Chen, Huiling Zhang, Ruixue Dou, Chaorui Wang, Muhammad Ishaq, Xiaoying Wei, Zitong Ding, Xusheng Guo
**Manuscript Type**	Research articles
**Total Character Count (including spaces)[1]**	49955
**Word count of Summary[2]**	201
**Number of papers cited in the References[3]**	45
**Listing of all Tables (Table1, Table 2 etc)[4]**	None

**Figure specifications (please complete one row per figure)[5]**<br><br>***Figure no.***	Colour<br>***(yes/no)***	Greyscale<br>***(yes/no)***	Black and white<br>***(yes/no)***	Single column (80mm)<br>***(yes/no)***	Double column (180mm)<br>***(yes/no)***	Size of figure at full scale (mm x mm)<br>***(insert details)***	Smallest font size used in the figure at full scale (minimum 6pt)<br>***(insert details)***
Figure 1	yes	no	no	no	yes	360 x 420	12.96
Figure 2	yes	no	no	no	yes	360 x 460	10
Figure 3	yes	no	no	no	yes	360 x 460	12
Figure 4	yes	no	no	no	yes	360 x 460	9.79
Figure 5	yes	no	no	no	yes	360 x 460	10.92
Figure 6	yes	no	no	no	yes	360 x 369	8

[1] The maximum character count allowed is 50,000 (incl. spaces) for Primary Research Papers and Reviews, 10,000 for Short Takes.

[2] Summary should not exceed 250 words.

[3] Primary Research Papers can contain a maximum of two tables. If more are needed they should replace some of the Figures or can be placed in the Supporting Information.

[4] A maximum of 45 references is allowed for Primary Research Papers and 20 references for Short Takes.

[5] A Primary Research Paper may contain up to 6 figures and a Short Take up to 2 figures. Authors are encouraged to provide figures in the size they are to appear in the journal and at the specifications given.
