# Supplementary material for: Lactiplantibacillus plantarum AS21 Attenuates Sex‐Biased Glucose Intolerance in Aging Mice via Gut Mucosal Microbiota‐Derived Citrulline
Source: Aging Cell. 2026 Aug 13;25(8):e70662. doi: 10.1111/acel.70662 (PMC13473181; doi:10.1111/acel.70662)
Supplement: Supplementary file 5 [file ACEL-25-e70662-s006.pdf]

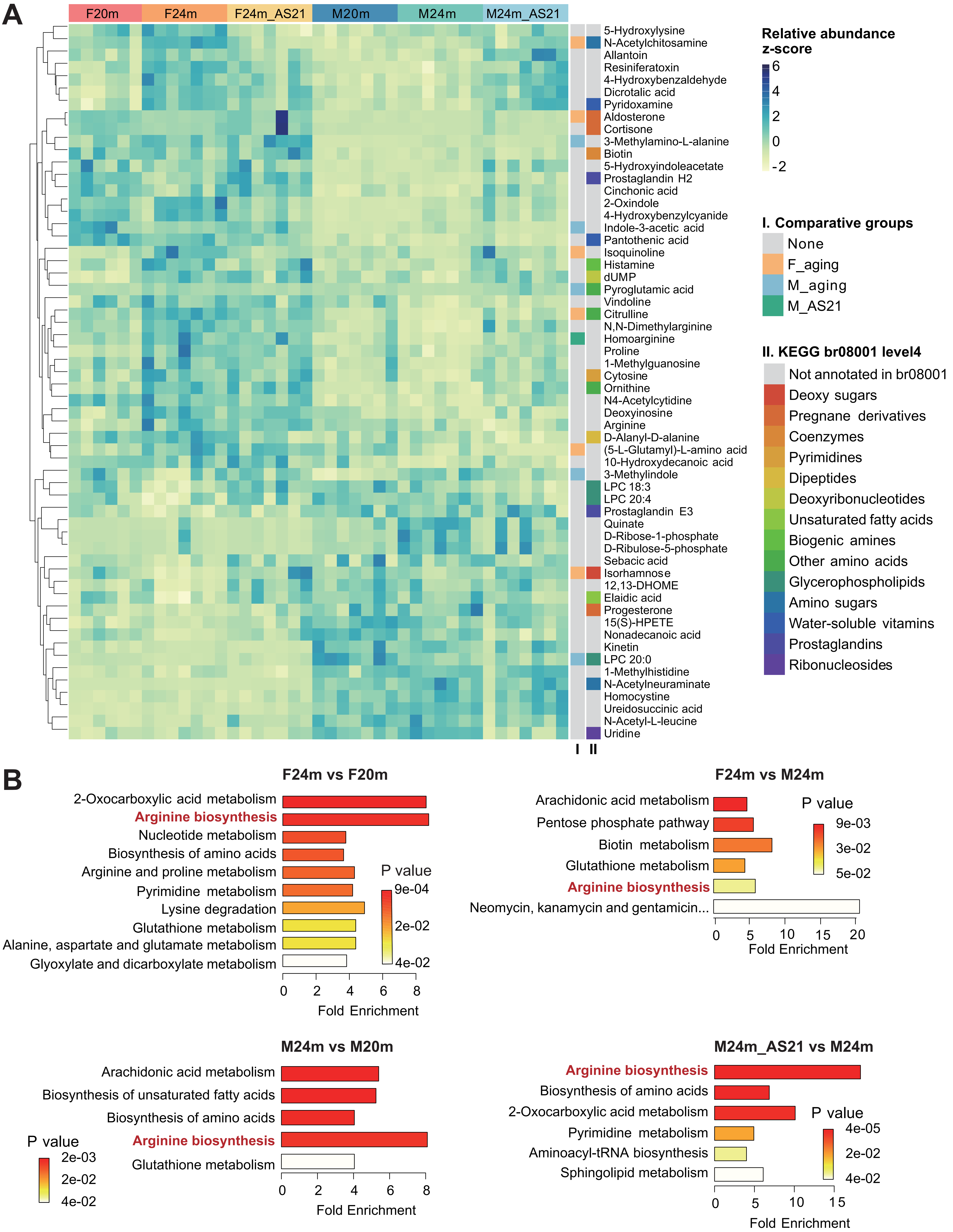

A
F20m
F24m
F24m_AS21
M20m
M24m
M24m_AS21
5-Hydroxylysine
N-Acetylchitosamine
Allantoin
Resiniferatoxin
4-Hydroxybenzaldehyde
Dicrotalic acid
Pyridoxamine
Aldosterone
Cortisone
3-Methylamino-L-alanine
Biotin
5-Hydroxyindoleacetate
Prostaglandin H2
Cinchonic acid
2-Oxindole
4-Hydroxybenzylcyanide
Indole-3-acetic acid
Pantothenic acid
Isoquinoline
Histamine
dUMP
Pyroglutamic acid
Vindoline
Citrulline
N,N-Dimethylarginine
Homoarginine
Proline
1-Methylguanosine
Cytosine
Ornithine
N4-Acetylcytidine
Deoxyinosine
Arginine
D-Alanyl-D-alanine
(5-L-Glutamyl)-L-amino acid
10-Hydroxydecanoic acid
3-Methylindole
LPC 18:3
LPC 20:4
Prostaglandin E3
Quinate
D-Ribose-1-phosphate
D-Ribulose-5-phosphate
Sebacic acid
Isorhamnose
12,13-DHOME
Elaidic acid
Progesterone
15(S)-HPETE
Nonadecanoic acid
Kinetin
LPC 20:0
1-Methylhistidine
N-Acetylneuraminate
Homocystine
Ureidosuccinic acid
N-Acetyl-L-leucine
Uridine
I II
Relative abundance z-score
6
4
2
0
-2
I. Comparative groups
None
F_aging
M_aging
M_AS21
II. KEGG br08001 level4
Not annotated in br08001
Deoxy sugars
Pregnane derivatives
Coenzymes
Pyrimidines
Dipeptides
Deoxyribonucleotides
Unsaturated fatty acids
Biogenic amines
Other amino acids
Glycerophospholipids
Amino sugars
Water-soluble vitamins
Prostaglandins
Ribonucleosides
B
F24m vs F20m
2-Oxocarboxylic acid metabolism
Arginine biosynthesis
Nucleotide metabolism
Biosynthesis of amino acids
Arginine and proline metabolism
Pyrimidine metabolism
Lysine degradation
Glutathione metabolism
Alanine, aspartate and glutamate metabolism
Glyoxylate and dicarboxylate metabolism
P value
9e-04
2e-02
4e-02
0 2 4 6 8
Fold Enrichment
F24m vs M24m
Arachidonic acid metabolism
Pentose phosphate pathway
Biotin metabolism
Glutathione metabolism
Arginine biosynthesis
Neomycin, kanamycin and gentamicin...
P value
9e-03
3e-02
5e-02
0 5 10 15 20
Fold Enrichment
M24m vs M20m
Arachidonic acid metabolism
Biosynthesis of unsaturated fatty acids
Biosynthesis of amino acids
Arginine biosynthesis
Glutathione metabolism
P value
2e-03
2e-02
4e-02
0 2 4 6 8
Fold Enrichment
M24m_AS21 vs M24m
Arginine biosynthesis
Biosynthesis of amino acids
2-Oxocarboxylic acid metabolism
Pyrimidine metabolism
Aminoacyl-tRNA biosynthesis
Sphingolipid metabolism
P value
4e-05
2e-02
4e-02
0 5 10 15
Fold Enrichment
