# Supplementary material for: Lactiplantibacillus plantarum AS21 Attenuates Sex‐Biased Glucose Intolerance in Aging Mice via Gut Mucosal Microbiota‐Derived Citrulline
Source: Aging Cell. 2026 Aug 13;25(8):e70662. doi: 10.1111/acel.70662 (PMC13473181; doi:10.1111/acel.70662)
Supplement: Supplementary file 6 [file ACEL-25-e70662-s008.pdf]

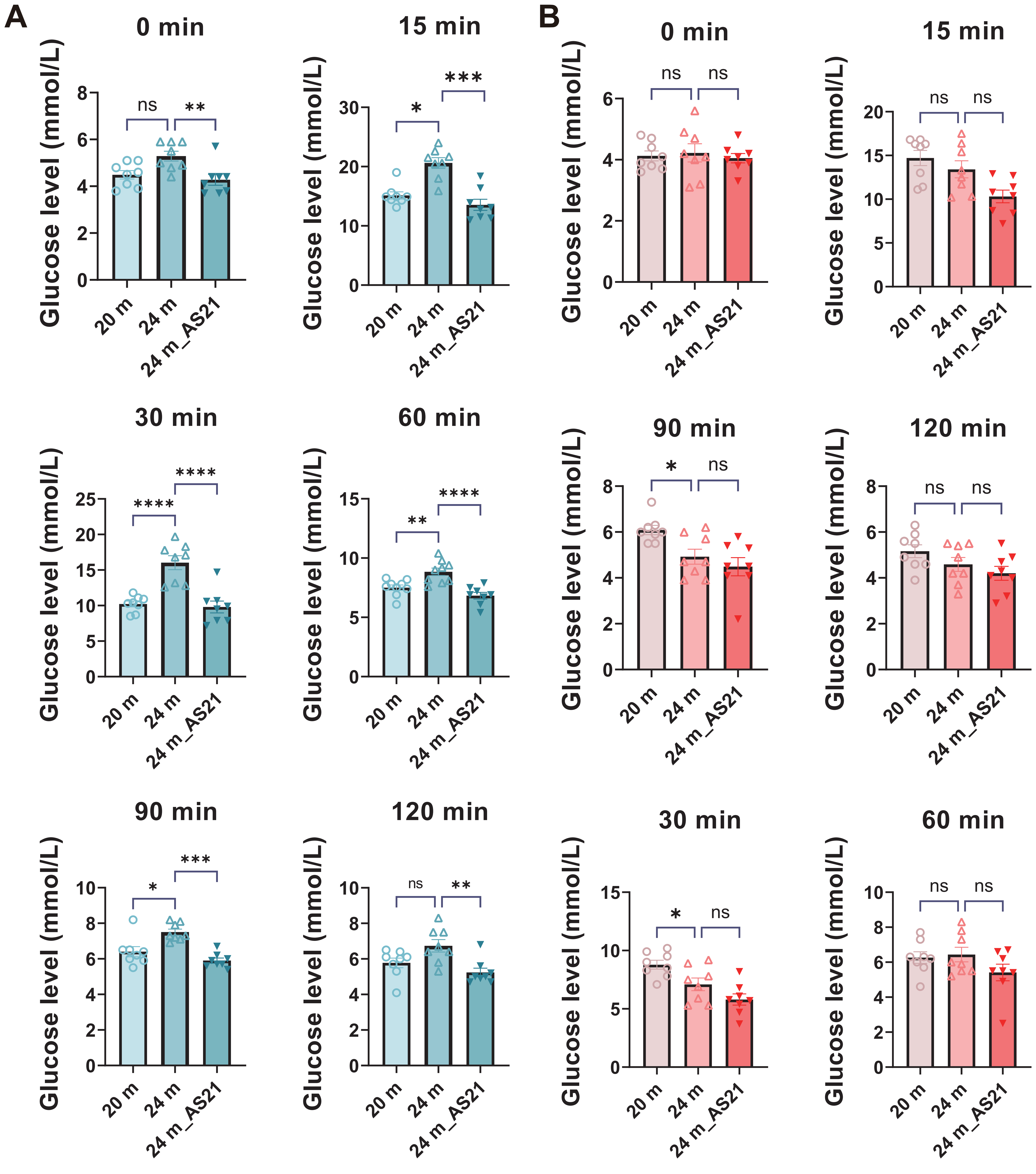
A
B
0 min
15 min
30 min
60 min
90 min
120 min
Glucose level (mmol/L)
20 m
24 m
24 m_AS21
ns
*
**
***
****
