# Supplementary material for: Lactiplantibacillus plantarum AS21 Attenuates Sex‐Biased Glucose Intolerance in Aging Mice via Gut Mucosal Microbiota‐Derived Citrulline
Source: Aging Cell. 2026 Aug 13;25(8):e70662. doi: 10.1111/acel.70662 (PMC13473181; doi:10.1111/acel.70662)
Supplement: Supplementary file 7 [file ACEL-25-e70662-s009.pdf]

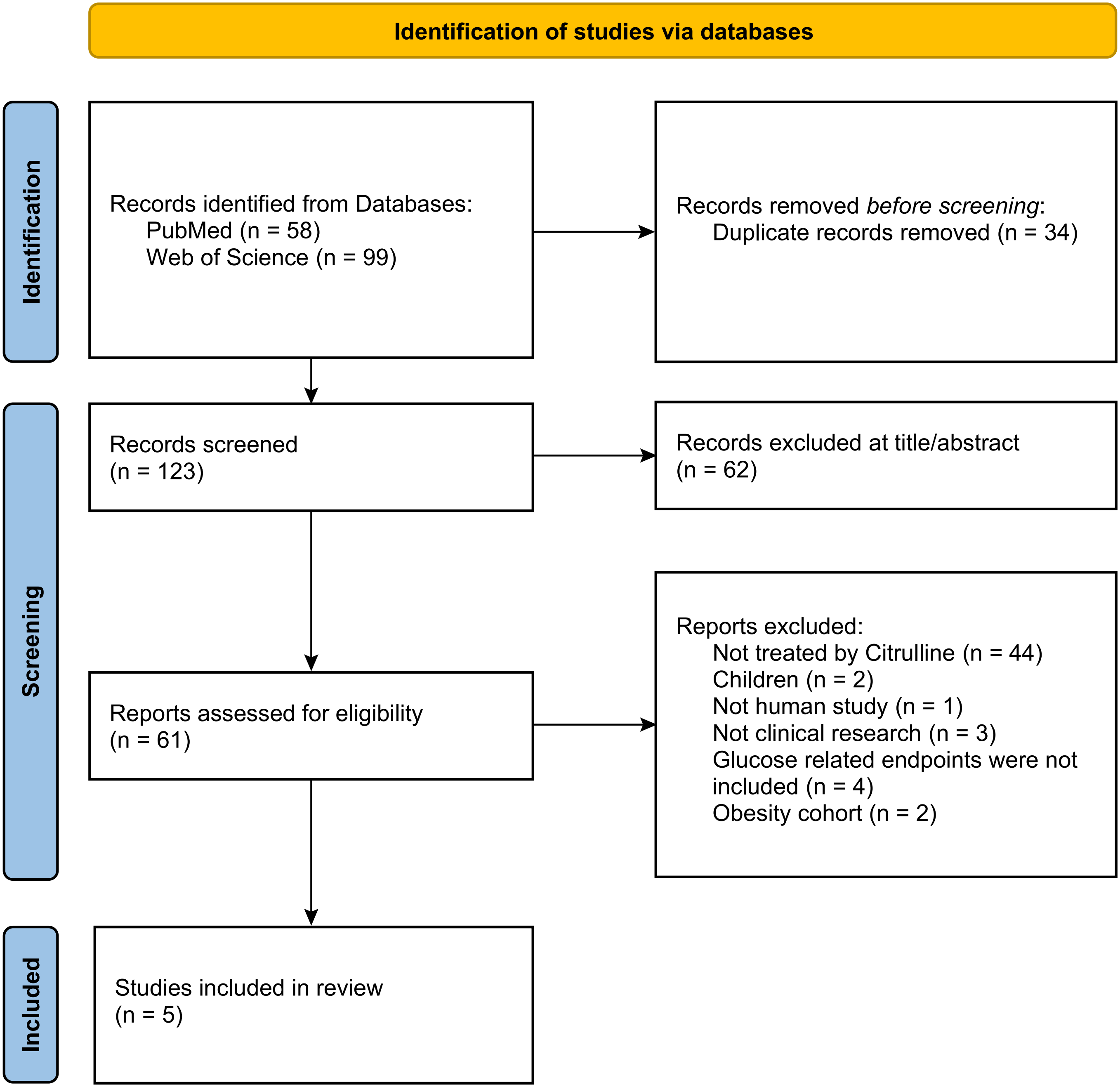
Identification of studies via databases
Identification
Records identified from Databases:
PubMed (n = 58)
Web of Science (n = 99)
Records removed *before screening*:
Duplicate records removed (n = 34)
Screening
Records screened
(n = 123)
Records excluded at title/abstract
(n = 62)
Reports assessed for eligibility
(n = 61)
Reports excluded:
Not treated by Citrulline (n = 44)
Children (n = 2)
Not human study (n = 1)
Not clinical research (n = 3)
Glucose related endpoints were not included (n = 4)
Obesity cohort (n = 2)
Included
Studies included in review
(n = 5)
