# Supplementary material for: Lactiplantibacillus plantarum AS21 Attenuates Sex‐Biased Glucose Intolerance in Aging Mice via Gut Mucosal Microbiota‐Derived Citrulline
Source: Aging Cell. 2026 Aug 13;25(8):e70662. doi: 10.1111/acel.70662 (PMC13473181; doi:10.1111/acel.70662)
Supplement: Supplementary file 8 [file ACEL-25-e70662-s010.pdf]

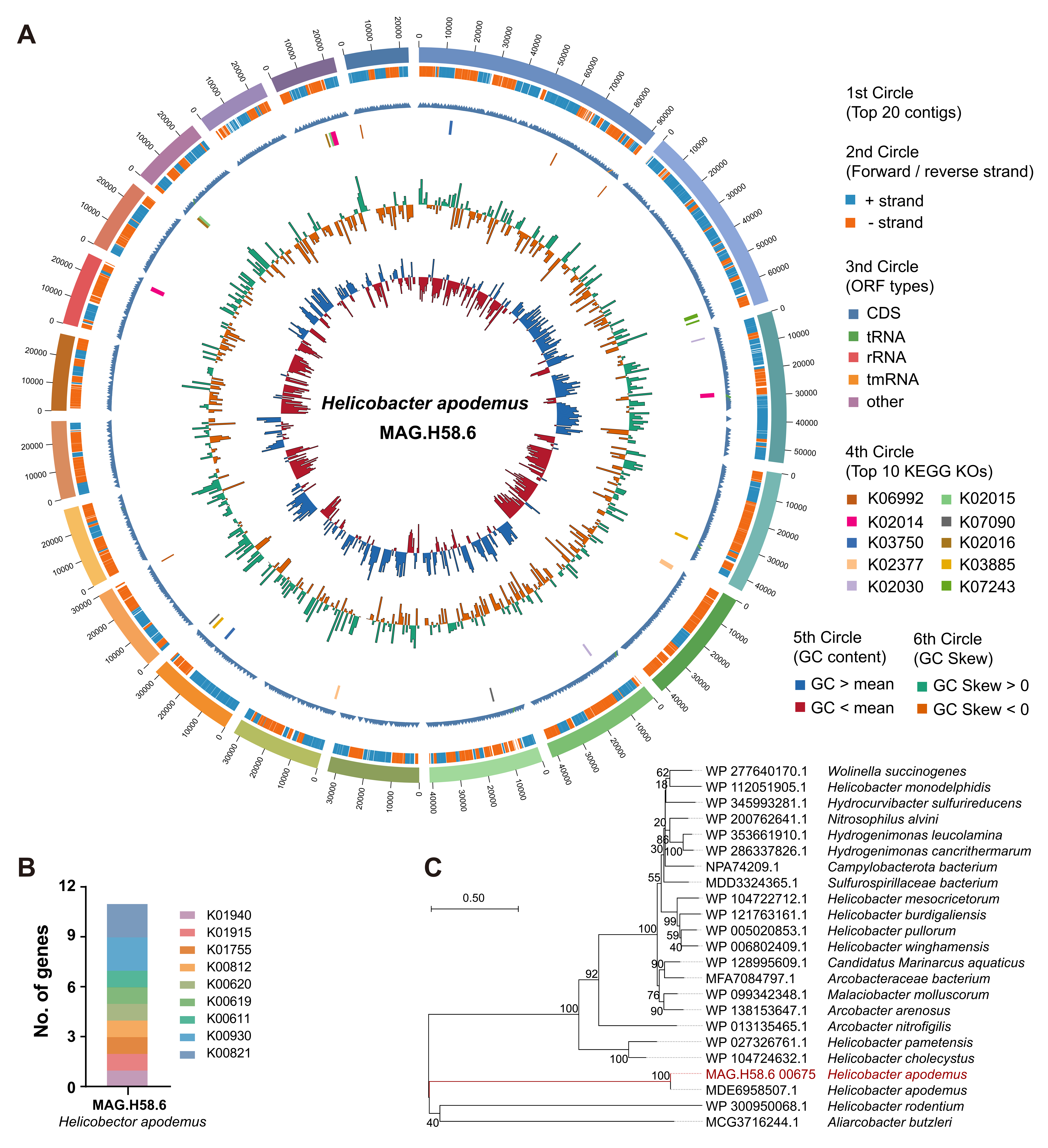

A
Helicobacter apodemus
MAG.H58.6
1st Circle
(Top 20 contigs)
2nd Circle
(Forward / reverse strand)
+ strand
- strand
3nd Circle
(ORF types)
CDS
tRNA
rRNA
tmRNA
other
4th Circle
(Top 10 KEGG KOs)
K06992
K02015
K02014
K07090
K03750
K02016
K02377
K03885
K02030
K07243
5th Circle
(GC content)
GC > mean
GC < mean
6th Circle
(GC Skew)
GC Skew > 0
GC Skew < 0
B
No. of genes
12
9
6
3
0
K01940
K01915
K01755
K00812
K00620
K00619
K00611
K00930
K00821
MAG.H58.6
Helicobector apodemus
C
0.50
WP 277640170.1 Wolinella succinogenes
WP 112051905.1 Helicobacter monodelphidis
WP 345993281.1 Hydrocurvibacter sulfurireducens
WP 200762641.1 Nitrosophilus alvini
WP 353661910.1 Hydrogenimonas leucolamina
WP 286337826.1 Hydrogenimonas cancrithermarum
NPA74209.1 Campylobacterota bacterium
MDD3324365.1 Sulfurospirillaceae bacterium
WP 104722712.1 Helicobacter mesocricetorum
WP 121763161.1 Helicobacter burdigaliensis
WP 005020853.1 Helicobacter pullorum
WP 006802409.1 Helicobacter winghamensis
WP 128995609.1 Candidatus Marinarcus aquaticus
MFA7084797.1 Arcobacteraceae bacterium
WP 099342348.1 Malaciobacter molluscorum
WP 138153647.1 Arcobacter arenosus
WP 013135465.1 Arcobacter nitrofigilis
WP 027326761.1 Helicobacter pametensis
WP 104724632.1 Helicobacter cholecystus
MAG.H58.6 00675 Helicobacter apodemus
MDE6958507.1 Helicobacter apodemus
WP 300950068.1 Helicobacter rodentium
MCG3716244.1 Aliarcobacter butzleri
