# Supplementary material for: Lactiplantibacillus plantarum AS21 Attenuates Sex‐Biased Glucose Intolerance in Aging Mice via Gut Mucosal Microbiota‐Derived Citrulline
Source: Aging Cell. 2026 Aug 13;25(8):e70662. doi: 10.1111/acel.70662 (PMC13473181; doi:10.1111/acel.70662)
Supplement: Supplementary file 9 [file ACEL-25-e70662-s011.pdf]

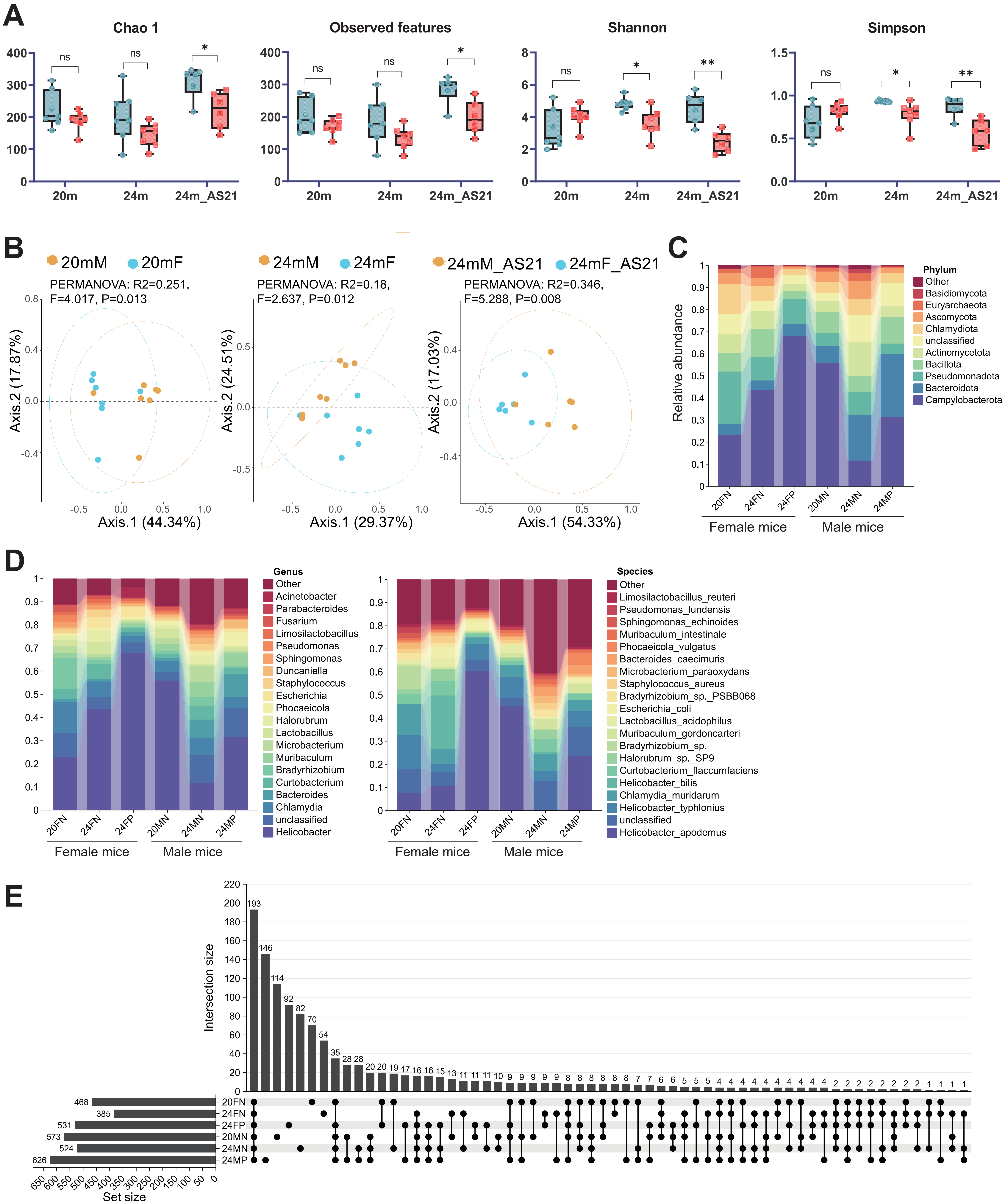

A
Chao 1
Observed features
Shannon
Simpson
ns
*
**
20m
24m
24m_AS21
B
20mM 20mF
PERMANOVA: R2=0.251, F=4.017, P=0.013
Axis.2 (17.87%)
Axis.1 (44.34%)
24mM 24mF
PERMANOVA: R2=0.18, F=2.637, P=0.012
Axis.2 (24.51%)
Axis.1 (29.37%)
24mM_AS21 24mF_AS21
PERMANOVA: R2=0.346, F=5.288, P=0.008
Axis.2 (17.03%)
Axis.1 (54.33%)
C
Relative abundance
Phylum
Other
Basidiomycota
Euryarchaeota
Ascomycota
Chlamydiota
unclassified
Actinomycetota
Bacillota
Pseudomonadota
Bacteroidota
Campylobacterota
20FN
24FN
24FP
20MN
24MN
24MP
Female mice
Male mice
D
Genus
Other
Acinetobacter
Parabacteroides
Fusarium
Limosilactobacillus
Pseudomonas
Sphingomonas
Duncaniella
Staphylococcus
Escherichia
Phocaeicola
Halorubrum
Lactobacillus
Microbacterium
Muribaculum
Bradyrhizobium
Curtobacterium
Bacteroides
Chlamydia
unclassified
Helicobacter
Species
Other
Limosilactobacillus_reuteri
Pseudomonas_lundensis
Sphingomonas_echinoides
Muribaculum_intestinale
Phocaeicola_vulgatus
Bacteroides_caecimuris
Microbacterium_paraoxydans
Staphylococcus_aureus
Bradyrhizobium_sp._PSBB068
Escherichia_coli
Lactobacillus_acidophilus
Muribaculum_gordoncarteri
Bradyrhizobium_sp.
Halorubrum_sp._SP9
Curtobacterium_flaccumfaciens
Helicobacter_bilis
Chlamydia_muridarum
Helicobacter_typhlonius
unclassified
Helicobacter_apodemus
E
Intersection size
Set size
468 20FN
385 24FN
531 24FP
573 20MN
524 24MN
626 24MP
