# Supplementary material for: Lactiplantibacillus plantarum AS21 Attenuates Sex‐Biased Glucose Intolerance in Aging Mice via Gut Mucosal Microbiota‐Derived Citrulline
Source: Aging Cell. 2026 Aug 13;25(8):e70662. doi: 10.1111/acel.70662 (PMC13473181; doi:10.1111/acel.70662)
Supplement: Supplementary file 10 [file ACEL-25-e70662-s012.pdf]

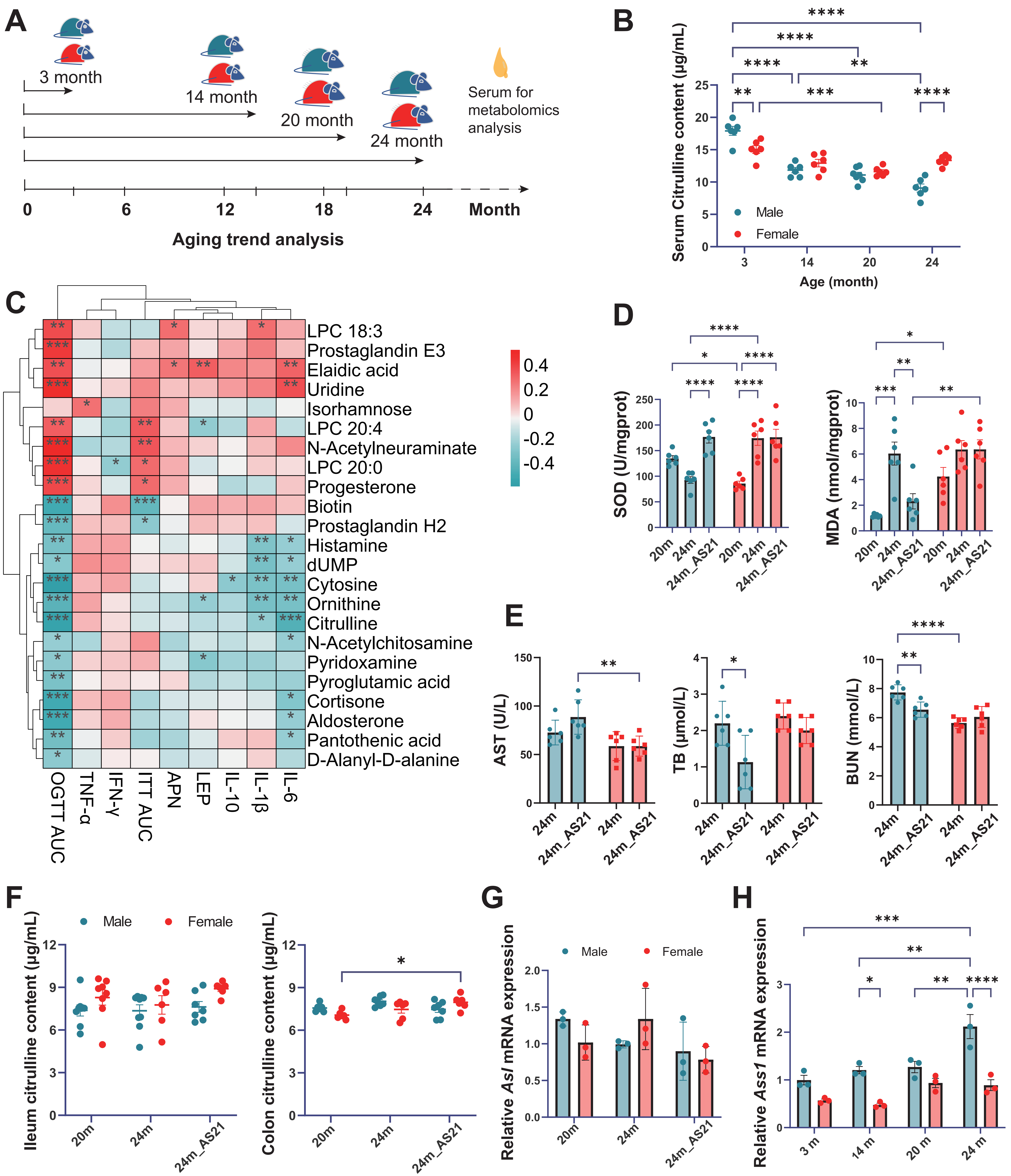

A
3 month
14 month
20 month
24 month
Serum for metabolomics analysis
0
6
12
18
24
Month
Aging trend analysis
B
Serum Citrulline content (μg/mL)
Age (month)
Male
Female
C
LPC 18:3
Prostaglandin E3
Elaidic acid
Uridine
Isorhamnose
LPC 20:4
N-Acetylneuraminate
LPC 20:0
Progesterone
Biotin
Prostaglandin H2
Histamine
dUMP
Cytosine
Ornithine
Citrulline
N-Acetylchitosamine
Pyridoxamine
Pyroglutamic acid
Cortisone
Aldosterone
Pantothenic acid
D-Alanyl-D-alanine
OGTT AUC
TNF-α
IFN-γ
ITT AUC
APN
LEP
IL-10
IL-1β
IL-6
D
SOD (U/mgprot)
MDA (nmol/mgprot)
20m
24m
24m_AS21
E
AST (U/L)
TB (μmol/L)
BUN (mmol/L)
F
Ileum citrulline content (μg/mL)
Colon citrulline content (μg/mL)
G
Relative *Asl* mRNA expression
H
Relative *Ass1* mRNA expression
3 m
14 m
20 m
24 m
