# Supplementary material for: Lactiplantibacillus plantarum AS21 Attenuates Sex‐Biased Glucose Intolerance in Aging Mice via Gut Mucosal Microbiota‐Derived Citrulline
Source: Aging Cell. 2026 Aug 13;25(8):e70662. doi: 10.1111/acel.70662 (PMC13473181; doi:10.1111/acel.70662)
Supplement: Supplementary file 11 [file ACEL-25-e70662-s013.pdf]

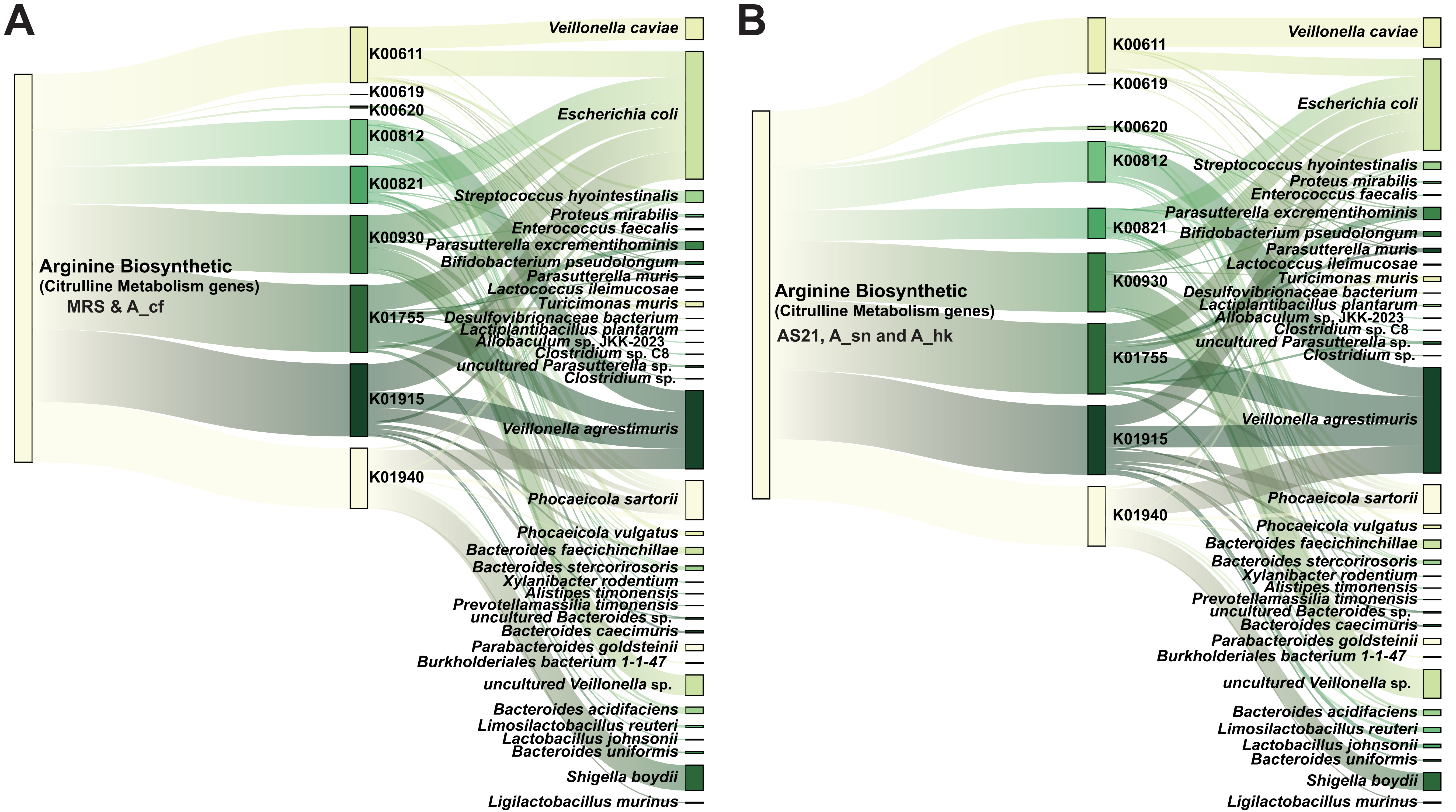

A
Arginine Biosynthetic
(Citrulline Metabolism genes)
MRS & A_cf
K00611
K00619
K00620
K00812
K00821
K00930
K01755
K01915
K01940
Veillonella caviae
Escherichia coli
Streptococcus hyointestinalis
Proteus mirabilis
Enterococcus faecalis
Parasutterella excrementihominis
Bifidobacterium pseudolongum
Parasutterella muris
Lactococcus ileimucosae
Turicimonas muris
Desulfovibrionaceae bacterium
Lactiplantibacillus plantarum
Allobaculum sp. JKK-2023
Clostridium sp. C8
uncultured Parasutterella sp.
Clostridium sp.
Veillonella agrestimuris
Phocaeicola sartorii
Phocaeicola vulgatus
Bacteroides faecichinchillae
Bacteroides stercorirosoris
Xylanibacter rodentium
Alistipes timonensis
Prevotellamassilia timonensis
uncultured Bacteroides sp.
Bacteroides caecimuris
Parabacteroides goldsteinii
Burkholderiales bacterium 1-1-47
uncultured Veillonella sp.
Bacteroides acidifaciens
Limosilactobacillus reuteri
Lactobacillus johnsonii
Bacteroides uniformis
Shigella boydii
Ligilactobacillus murinus
B
Arginine Biosynthetic
(Citrulline Metabolism genes)
AS21, A_sn and A_hk
K00611
K00619
K00620
K00812
K00821
K00930
K01755
K01915
K01940
Veillonella caviae
Escherichia coli
Streptococcus hyointestinalis
Proteus mirabilis
Enterococcus faecalis
Parasutterella excrementihominis
Bifidobacterium pseudolongum
Parasutterella muris
Lactococcus ileimucosae
Turicimonas muris
Desulfovibrionaceae bacterium
Lactiplantibacillus plantarum
Allobaculum sp. JKK-2023
Clostridium sp. C8
uncultured Parasutterella sp.
Clostridium sp.
Veillonella agrestimuris
Phocaeicola sartorii
Phocaeicola vulgatus
Bacteroides faecichinchillae
Bacteroides stercorirosoris
Xylanibacter rodentium
Alistipes timonensis
Prevotellamassilia timonensis
uncultured Bacteroides sp.
Bacteroides caecimuris
Parabacteroides goldsteinii
Burkholderiales bacterium 1-1-47
uncultured Veillonella sp.
Bacteroides acidifaciens
Limosilactobacillus reuteri
Lactobacillus johnsonii
Bacteroides uniformis
Shigella boydii
Ligilactobacillus murinus
