# Supplementary material for: Lactiplantibacillus plantarum AS21 Attenuates Sex‐Biased Glucose Intolerance in Aging Mice via Gut Mucosal Microbiota‐Derived Citrulline
Source: Aging Cell. 2026 Aug 13;25(8):e70662. doi: 10.1111/acel.70662 (PMC13473181; doi:10.1111/acel.70662)
Supplement: Supplementary file 12 [file ACEL-25-e70662-s015.pdf]

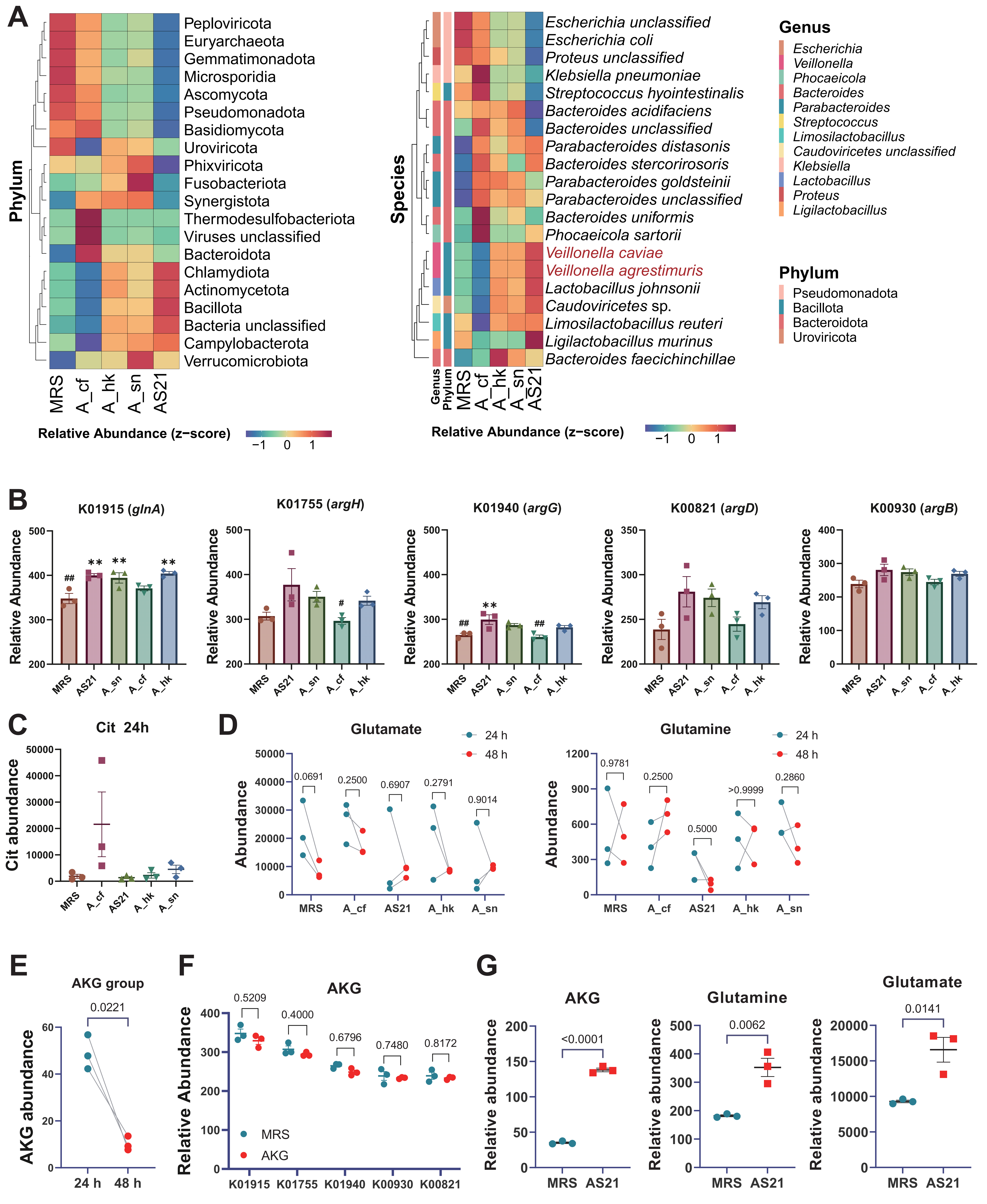
A
Phylum
Peploviricota
Euryarchaeota
Gemmatimonadota
Microsporidia
Ascomycota
Pseudomonadota
Basidiomycota
Uroviricota
Phixviricota
Fusobacteriota
Synergistota
Thermodesulfobacteriota
Viruses unclassified
Bacteroidota
Chlamydiota
Actinomycetota
Bacillota
Bacteria unclassified
Campylobacterota
Verrucomicrobiota
MRS
A_cf
A_hk
A_sn
AS21
Relative Abundance (z−score)
−1
0
1
Species
Escherichia unclassified
Escherichia coli
Proteus unclassified
Klebsiella pneumoniae
Streptococcus hyointestinalis
Bacteroides acidifaciens
Bacteroides unclassified
Parabacteroides distasonis
Bacteroides stercorirosoris
Parabacteroides goldsteinii
Parabacteroides unclassified
Bacteroides uniformis
Phocaeicola sartorii
Veillonella caviae
Veillonella agrestimuris
Lactobacillus johnsonii
Caudoviricetes sp.
Limosilactobacillus reuteri
Ligilactobacillus murinus
Bacteroides faecichinchillae
Genus
Phylum
MRS
A_cf
A_hk
A_sn
AS21
Relative Abundance (z−score)
−1
0
1
Genus
Escherichia
Veillonella
Phocaeicola
Bacteroides
Parabacteroides
Streptococcus
Limosilactobacillus
Caudoviricetes unclassified
Klebsiella
Lactobacillus
Proteus
Ligilactobacillus
Phylum
Pseudomonadota
Bacillota
Bacteroidota
Uroviricota
B
K01915 (glnA)
K01755 (argH)
K01940 (argG)
K00821 (argD)
K00930 (argB)
Relative Abundance
MRS
AS21
A_sn
A_cf
A_hk
##
**
#
C
Cit 24h
Cit abundance
MRS
A_cf
AS21
A_hk
A_sn
D
Glutamate
Glutamine
24 h
48 h
Abundance
0.0691
0.2500
0.6907
0.2791
0.9014
0.9781
0.2500
0.5000
>0.9999
0.2860
MRS
A_cf
AS21
A_hk
A_sn
E
AKG group
AKG abundance
0.0221
24 h
48 h
F
AKG
Relative Abundance
0.5209
0.4000
0.6796
0.7480
0.8172
MRS
AKG
K01915
K01755
K01940
K00930
K00821
G
AKG
Glutamine
Glutamate
Relative abundance
<0.0001
0.0062
0.0141
MRS
AS21
