# Supplementary material for: Lactiplantibacillus plantarum AS21 Attenuates Sex‐Biased Glucose Intolerance in Aging Mice via Gut Mucosal Microbiota‐Derived Citrulline
Source: Aging Cell. 2026 Aug 13;25(8):e70662. doi: 10.1111/acel.70662 (PMC13473181; doi:10.1111/acel.70662)
Supplement: Supplementary file 13 [file ACEL-25-e70662-s016.pdf]

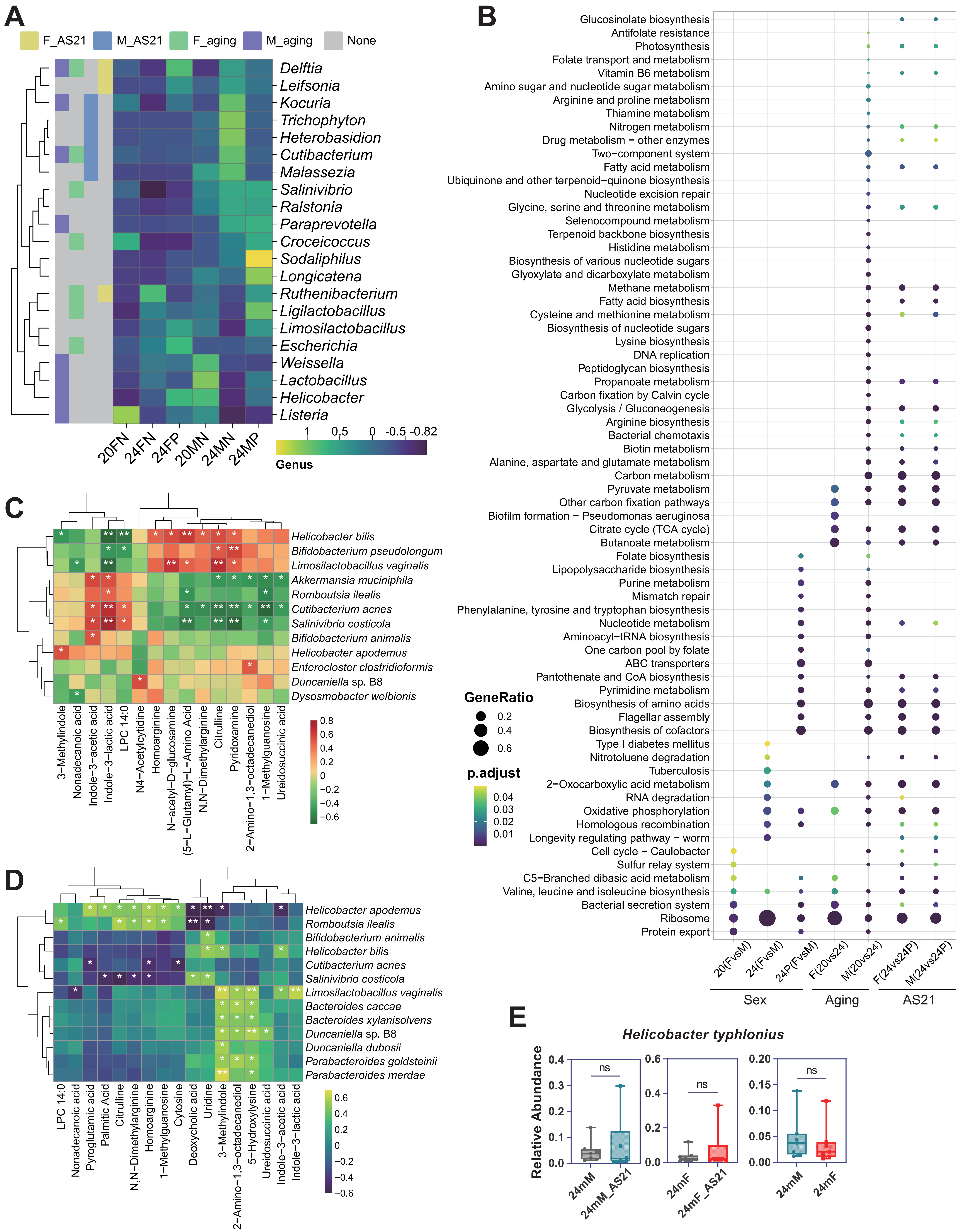
A
F_AS21
M_AS21
F_aging
M_aging
None
Delftia
Leifsonia
Kocuria
Trichophyton
Heterobasidion
Cutibacterium
Malassezia
Salinivibrio
Ralstonia
Paraprevotella
Croceicoccus
Sodaliphilus
Longicatena
Ruthenibacterium
Ligilactobacillus
Limosilactobacillus
Escherichia
Weissella
Lactobacillus
Helicobacter
Listeria
20FN
24FN
24FP
20MN
24MN
24MP
1
0.5
0
-0.5
-0.82
Genus
B
Glucosinolate biosynthesis
Antifolate resistance
Photosynthesis
Folate transport and metabolism
Vitamin B6 metabolism
Amino sugar and nucleotide sugar metabolism
Arginine and proline metabolism
Thiamine metabolism
Nitrogen metabolism
Drug metabolism − other enzymes
Two−component system
Fatty acid metabolism
Ubiquinone and other terpenoid−quinone biosynthesis
Nucleotide excision repair
Glycine, serine and threonine metabolism
Selenocompound metabolism
Terpenoid backbone biosynthesis
Histidine metabolism
Biosynthesis of various nucleotide sugars
Glyoxylate and dicarboxylate metabolism
Methane metabolism
Fatty acid biosynthesis
Cysteine and methionine metabolism
Biosynthesis of nucleotide sugars
Lysine biosynthesis
DNA replication
Peptidoglycan biosynthesis
Propanoate metabolism
Carbon fixation by Calvin cycle
Glycolysis / Gluconeogenesis
Arginine biosynthesis
Bacterial chemotaxis
Biotin metabolism
Alanine, aspartate and glutamate metabolism
Carbon metabolism
Pyruvate metabolism
Other carbon fixation pathways
Biofilm formation − Pseudomonas aeruginosa
Citrate cycle (TCA cycle)
Butanoate metabolism
Folate biosynthesis
Lipopolysaccharide biosynthesis
Purine metabolism
Mismatch repair
Phenylalanine, tyrosine and tryptophan biosynthesis
Nucleotide metabolism
Aminoacyl−tRNA biosynthesis
One carbon pool by folate
ABC transporters
Pantothenate and CoA biosynthesis
Pyrimidine metabolism
Biosynthesis of amino acids
Flagellar assembly
Biosynthesis of cofactors
Type I diabetes mellitus
Nitrotoluene degradation
Tuberculosis
2−Oxocarboxylic acid metabolism
RNA degradation
Oxidative phosphorylation
Homologous recombination
Longevity regulating pathway − worm
Cell cycle − Caulobacter
Sulfur relay system
C5−Branched dibasic acid metabolism
Valine, leucine and isoleucine biosynthesis
Bacterial secretion system
Ribosome
Protein export
GeneRatio
0.2
0.4
0.6
p.adjust
0.04
0.03
0.02
0.01
20(FvsM)
24(FvsM)
24P(FvsM)
F(20vs24)
M(20vs24)
F(24vs24P)
M(24vs24P)
Sex
Aging
AS21
C
Helicobacter bilis
Bifidobacterium pseudolongum
Limosilactobacillus vaginalis
Akkermansia muciniphila
Romboutsia ilealis
Cutibacterium acnes
Salinivibrio costicola
Bifidobacterium animalis
Helicobacter apodemus
Enterocloster clostridioformis
Duncaniella sp. B8
Dysosmobacter welbionis
3−Methylindole
Nonadecanoic acid
Indole−3−acetic acid
Indole−3−lactic acid
LPC 14:0
N4−Acetylcytidine
Homoarginine
N−acetyl−D−glucosamine
(5−L−Glutamyl)−L−Amino Acid
N,N−Dimethylarginine
Citrulline
Pyridoxamine
2−Amino−1,3−octadecanediol
1−Methylguanosine
Ureidosuccinic acid
0.8
0.6
0.4
0.2
0
−0.2
−0.4
−0.6
D
Helicobacter apodemus
Romboutsia ilealis
Bifidobacterium animalis
Helicobacter bilis
Cutibacterium acnes
Salinivibrio costicola
Limosilactobacillus vaginalis
Bacteroides caccae
Bacteroides xylanisolvens
Duncaniella sp. B8
Duncaniella dubosii
Parabacteroides goldsteinii
Parabacteroides merdae
LPC 14:0
Nonadecanoic acid
Pyroglutamic acid
Palmitic Acid
Citrulline
N,N−Dimethylarginine
Homoarginine
1−Methylguanosine
Cytosine
Deoxycholic acid
Uridine
3−Methylindole
2−Amino−1,3−octadecanediol
5−Hydroxylysine
Ureidosuccinic acid
Indole−3−acetic acid
Indole−3−lactic acid
0.6
0.4
0.2
0
−0.2
−0.4
−0.6
E
Helicobacter typhlonius
Relative Abundance
ns
ns
ns
24mM
24mM_AS21
24mF
24mF_AS21
24mM
24mF
