# Supplementary material for: Lactiplantibacillus plantarum AS21 Attenuates Sex‐Biased Glucose Intolerance in Aging Mice via Gut Mucosal Microbiota‐Derived Citrulline
Source: Aging Cell. 2026 Aug 13;25(8):e70662. doi: 10.1111/acel.70662 (PMC13473181; doi:10.1111/acel.70662)
Supplement: Supplementary file 14 [file ACEL-25-e70662-s018.pdf]

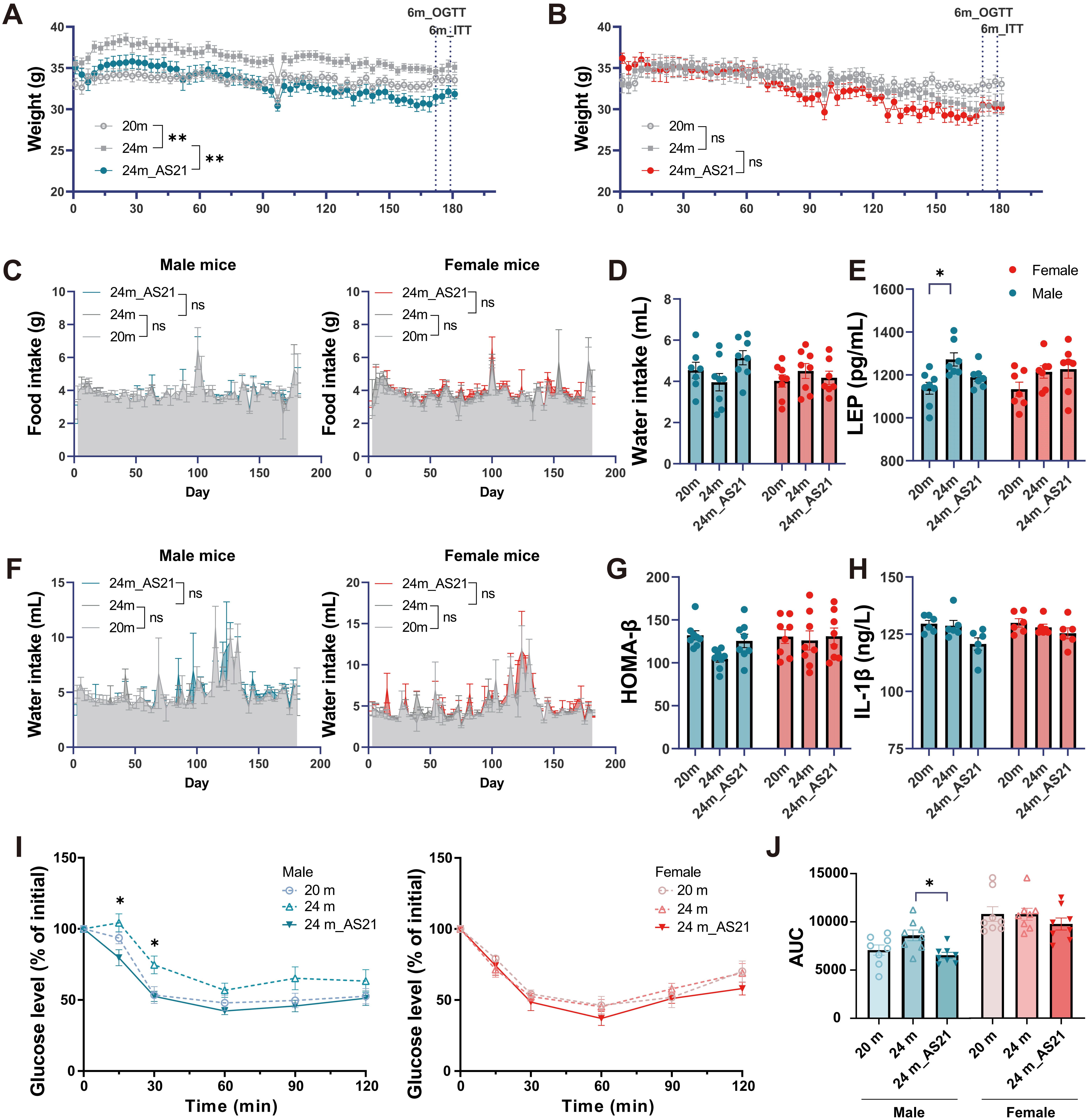

A
B
6m_OGTT
6m_ITT
Weight (g)
20m
24m
24m_AS21
**
ns
Male mice
Female mice
Food intake (g)
Day
D
Water intake (mL)
E
LEP (pg/mL)
Female
Male
F
G
HOMA-β
H
IL-1β (ng/L)
I
Glucose level (% of initial)
Time (min)
20 m
24 m
24 m_AS21
J
AUC
*
