# Supplementary material for: Lactiplantibacillus plantarum AS21 Attenuates Sex‐Biased Glucose Intolerance in Aging Mice via Gut Mucosal Microbiota‐Derived Citrulline
Source: Aging Cell. 2026 Aug 13;25(8):e70662. doi: 10.1111/acel.70662 (PMC13473181; doi:10.1111/acel.70662)
Supplement: Supplementary file 15 [file ACEL-25-e70662-s003.docx]

**Table S2 Primer sequences for the qPCR**

| **Gene** | **Sequence** | **Product length** |
| --- | --- | --- |
| *Gapdh* | ATTGTCAGCAATGCATCCTG | 102 bp |
|  | ATGGACTGTGGTCATGAGCC |  |
| *Ass1* | CGTGAAGGGGCCAAGTATGT | 115 bp |
|  | TCCAGGGAGCGATGACCTTA |  |
| *Otc* | ACCAGGTTACGATGAAGACTGCC | 199 bp |
|  | AAGAGTGATCCAAGGCAAGGGG |  |
| *Asl* | ACATCCACACAGCCAACGAG | 199 bp |
|  | ACATCACGTTCCGCCTCTG |  |
| *Gcg* | AGACAGAAGCGCATGAGGAC | 159 bp |
|  | TGGCAATGTTGTTCCGGTTC |  |
| *Slc6a14* | AGCAATGAAACGCATGAGGG | 99 bp |
|  | ACCCAGTAAATTCCAGCCTGAG |  |
