# Supplementary material for: Lactiplantibacillus plantarum AS21 Attenuates Sex‐Biased Glucose Intolerance in Aging Mice via Gut Mucosal Microbiota‐Derived Citrulline
Source: Aging Cell. 2026 Aug 13;25(8):e70662. doi: 10.1111/acel.70662 (PMC13473181; doi:10.1111/acel.70662)
Supplement: Supplementary file 16 [file ACEL-25-e70662-s007.docx]

| **Table S1 Summary of clinical studies investigating citrulline supplementation and glycemic outcomes** | | | | | | | | |
| --- | --- | --- | --- | --- | --- | --- | --- | --- |
| **Author** | **Subjects and Study Models** | **Sample size  (Cit / Ctrl)** | **Age**  **(mean ± SD)** | **Sex**  **(% male)** | **BMI**  **(mean ± SD)** | **Dose**  **(g/day)** | **Duration (weeks)** | **Main Outcomes** |
| Alia Shatanawi et al (2020) (Shatanawi et al. 2020) | T2D patients, single-arm supplementation study, pre-post design | 25/0 | 42.7 ± 10.7 | 52% | unknown | 2 | 4 | HbA1c and FBG were not significantly changed before and after intervention |
| Yejin Kang et al (2025) (Kang et al. 2025) | T2D patients,  double-blind, randomized, placebo-controlled, crossover design | 16 | 62 ± 6 | 43.75% | 33.4 ± 4.9 | 6 | 4 | HbA1c, FBG, and insulin were significantly downregulated after intervention; HOMA-IR was not affected |
| Samaneh Azizi et al (2020a) (Azizi, Mahdavi, et al. 2021) Samaneh Azizi et al (2020b) (Azizi, Ebrahimi-Mameghani, et al. 2021) Fatemeh Abbaszadeh (2021) (Abbaszadeh et al. 2021) | T2D patients,  double-blind, randomized, placebo-controlled | 23/22 | 47.57 ± 5.60 / 49.86 ± 4.88 Cit / Ctrl | 60.90% / 68.20% Cit / Ctrl | 29.77 ± 3.20 / 28.26 ± 2.14 Cit / Ctrl | 3 | 8 | HbA1c, FBG, insulin, and HOMA-IR were significantly downregulated after intervention; HOMA-β was not affected by Cit treatment, but was upregulated in the Ctrl group |

Abbreviations: Cit, citrulline; Ctrl, control; SD, standard deviation.

**Reference**

Abbaszadeh, F., Azizi, S., Mobasseri, M., and Ebrahimi-Mameghani, M. 2021. "The effects of citrulline supplementation on meta-inflammation and insulin sensitivity in type 2 diabetes: a randomized, double-blind, placebo-controlled trial." *Diabetology & Metabolic Syndrome* 13, no. 1: 52. https://doi.org/10.1186/s13098-021-00669-w.

Azizi, S., Ebrahimi-Mameghani, M., Mobasseri, M., Karamzad, N., and Mahdavi, R. 2021. "Oxidative stress and nitrate/nitrite (NOx) status following citrulline supplementation in type 2 diabetes: a randomised, double-blind, placebo-controlled trial." *Journal of Human Nutrition and Dietetics* 34, no. 1: 64-72. https://doi.org/10.1111/jhn.12792.

Azizi, S., Mahdavi, R., Mobasseri, M., et al. 2021. "The impact of L-citrulline supplementation on glucose homeostasis, lipid profile, and some inflammatory factors in overweight and obese patients with type 2 diabetes: A double-blind randomized placebo-controlled trial." *Phytotherapy Research* 35, no. 6: 3157-3166. https://doi.org/10.1002/ptr.6997.

Kang, Y., Dillon, K. N., Levitt, D. E., and Figueroa, A. 2025. "Effects of L-citrulline supplementation on endothelial function, arterial stiffness, and blood glucose level in the fasted and acute hyperglycemic states in middle-aged and older adults with type 2 diabetes." *Nutrients* 17, no. 23: 3739. https://doi.org/10.3390/nu17233739.

Shatanawi, A., Momani, M. S., Al-Aqtash, R., Hamden, M. H., and Gharaibeh, M. N. 2020. "L-citrulline supplementation increases plasma nitric oxide levels and reduces arginase activity in patients with type 2 diabetes." *Frontiers in Pharmacology* 11, no.: 584669. https://doi.org/10.3389/fphar.2020.584669.
