# Supplementary material for: Lactiplantibacillus plantarum AS21 Attenuates Sex‐Biased Glucose Intolerance in Aging Mice via Gut Mucosal Microbiota‐Derived Citrulline
Source: Aging Cell. 2026 Aug 13;25(8):e70662. doi: 10.1111/acel.70662 (PMC13473181; doi:10.1111/acel.70662)
Supplement: Supplementary file 17 [file ACEL-25-e70662-s014.docx]

**Table S3 Protein sequence for ArgF used in Phylogenetic tree**

| **ID** | **Species** | **Sequence** |
| --- | --- | --- |
| MAG.H58.6_00675 | *Helicobacter apodemus* | INGLTDDFHPMQLLADFFTMQECHKDKNPIVAYIGDGNNMAHSWLMLAAKLGFELRIASPKNYQVSPKILQQAQAFAKISGSKLLIMQDPKEAILNADVVTTDTWTSMGQEKDKEMRKKAFKGYCVDKALLSLAKQDSIFLHCLPAYRGEEVTQEVLEGTQSKVFLEAENRLHIQKGVMVWLHQNR |
| WP_104722712.1 | *Helicobacter mesocricetorum* | MRHFLTLADFSKEEILEILQISKKLKKELKEGQYSNLLENKTLGMIFEKSSTRTRVSFEVGIHQLGGKGIFLSSNDIQLSRGEPIKDTARVLSSMVDMIMMRTFKHSSLEEFAKYSSVPVINGLTDDFHPMQLLADFFTMQECNKDQNPIVAYIGDGNNMAHSWLMLAAKLGFELRIASPKNYQVLPEILQKAQEFAKISGSKLLIMEDPKEAVLNADVVTTDTWASMGQEKDKKQRKKEFKDYCVNKSLLSLAKPDSIFLHCLPAYRGEEVTQEVLEGTQSKVFLEAENRLHTQKGVMVWLHQNR |
| MDE6958507.1 | *Helicobacter apodemus* | INGLTDDFHPMQLLADFFTMQECHKDKNPIVAYIGDGNNMAHSWLMLAAKLGFELRIASPKNYQVSPKILQQAQAFAKISGSKLLIMQDPKEAILNADVVTTDTWTSMGQEKDKEMRKKAFKGYCVDKALLSLAKQDSIFLHCLPAYRGEEVTQEVLEGTQSKVFLEAENRLHIQKGVMVWLHQNR |
| WP_005020853.1 | *Helicobacter pullorum* | MRHFLTLADFSKEEILEILQIAATLKKDLKAGKTSNLLEKKTLGMIFEKNSTRTRVSFETGIYQLGGQGIFLSSNDTQLGRGEPIKDTARVLSSMVDLIMMRTHEHSRLEEFAHYSKIPVINGLSDSFHPMQLLADYLTMQECQKDQNPIVAYIGDGNNMAHSWLMLAAKLGFELRIASPKGYEVCQKIFQKAQEFAKISGAKLILTQNPQEAVLNADVITTDTWASMGQEREKETRKKAFEGYCVDKNLMNLSKKDSIFLHCLPAYRGQEVSEEVLESTQSKIFLEAENRLHAQKGVMVWLHQNR |
| **ID** | **Species** | **Sequence**  **Table S3 (continued)** |
| WP_006802409.1 | *Helicobacter winghamensis* | MRHFLTLADFSKIEILEILKIAKALKEEVKSKKYSTALANQTLGMIFEKNSTRTRVSFETGIYQLGGQGIFLSSNDTQLGRGEPIKDTARVLSSMVDLIMMRTHGHDRLEEFAHYSSVPVINGLSDDFHPMQLLADYLTMQECGKDKNPIVAYIGDGNNMAHSWLMLASKLGFELRIASPKDYTISSKILEKAHEFAKVSGAKIIISEDPKAVAINADVITTDTWASMGQEDEKEARKKAFKDYCVDNTLMRLAKDDAIFLHCLPAYRGQEVSEEVLESHQSKIFQEAENRLHAQKGVMVWLHQNR |
| WP_300950068.1 | *Helicobacter rodentium* | GIYQLGGNGIFLSSNDTQLGRGEPIKDTARVLSSMVDLVMMRTHEHSRLEEFAKYSKVPVINGLSDDFHPMQLLADFMTMQECGKDKNPIVAYIGDGNNMAHSWLMLTSKLGFTLRIASPKGYEVSKEILKKAESFARISGAKILLSNNPREAVAGADVVTTDTWASMGQEEQKDKRKRDFKDYCVDSELMGLAKSDSIFLHCLPAYRGQEVSEEVLESNQSKIFQEAENRLHIQKGVMVWLHKSR |
| WP_121763161.1 | *Helicobacter burdigaliensis* | MRHLLTLADFSKEEILEILEIAKNLKTELKKGFYSKPLENKTLGMIFEKNSTRTRVSFEAGIYQLGGCGIFLSSNDTQLSRGEPIKDTARVLSSMVDLIMMRTSDHARLEEFAHYSKVPLINGLSDDFHPTQLLADYLTMQECGKDKDPIVAYIGDGNNMAHSWLMLASKMGFTLKIASPKGYEVKNEIYKQALEFAKISGAKISLTQDPKVAIKDADVVTTDTWASMGQENEKKERIEAFRGYCVNSELLALSKKDSIFLHCLPAYRGQEVSEEVLESPQSKIFIEAENRLHAQKGAMVWLHQNR |
| WP_128995609.1 | *Candidatus Marinarcus aquaticus* | MKHFLTLADFTKEELEEILALSIKVKQETKQRIFKDYLPKQVLGMIFEKSSTRTRVSFETGIYQLGGVGLFLSSSDIQLGRGEPMSDTSRVISRMVDMVMIRTFAHSKLEEFAQYSKVPVINGLTDDFHPVQLMADYMTIIEEGKDKDLVVAYVGDGNNLAHSWLMLASKLGFELRIATPKGYEVKEEILNKALGFAKESGAKIIVNNNPKEAVKNATVVTTDTWVSMGQEDEKEKRVKEFEGYIVDEAMMALAHKDAIFLHCLPAYRGYEVSEAVFEGSQSKVFEEAENRLHAQKGIMVWLNEKRND |
| **ID** | **Species** | **Sequence**  **Table S3 (continued)** |
| WP_277640170.1 | *Wolinella succinogenes* | MRHFLTLKDFSAQEILEILALSKEIKDDLKKGIRPPLMQGKVLGMIFEKSSTRTRVSFESGIYQLGGMGMFLSNRDIQLGRGEPMKDTARVISRMVDMVMIRTSGHERIEEFAKYSQVPVINGLTDSYHPVQLMADYLTMMEQGKDQNPIVAYVGDGNNMTHSWLMLASKLGFELRVATPRGYEPNLKIFEEAQKIASLSGAKITLMNDPKRAVEGADVVTTDTWASMGQEEEKEKRVRDFQGYCVDSALMGLAKSDAIFLHCLPAYRGQEVSEEVLEGPQSLVFDEAENRLHVQKGIMVWLDRKRS |
| WP_027326761.1 | *Helicobacter pametensis* | MKDFLSLKDYSKEEILQIIAIAQNIKQQHKSSIPTPLMKDCTLAMIFEKNSTRTRVSFETGIYQLGGIGLFLSANEIQLSRGEPIKDTARVIGSMVDMVMIRTFEHSRLEEFAAYCPVPVINGLSDSFHPVQLLADYLTMIECGISLDDQKPIVAYVGDGNNMAHSWLMLASKLGFELRIASPKGYGVDLAIMSLAQGFAQDSGAKITLLEDPKEAVSGANVVTTDTWASMGQEDEKEARVKAFDGYQVDCKMMSLAQKDAIFLHCLPAYRGYEVSEEVLEGGQSRVFLEAENRLHAQKGIMVYLHQNYKGNGC |
| WP_013135465.1 | *Arcobacter nitrofigilis* | MRHFLTIADFSKEELLEILDLAIQIKAETKQRNFKEYLPKQVLGMIFEKSSTRTRVSFETGIYQLGGIGLFLSSNDIQLGRGEPMSDTSRVISRMVDMVMIRTFEHSKLEEFAQYSKVPVINGLTDDFHPVQLLADYLTIIEEKDKDLVAAYVGDGNNLAHSWLMLAAKLGFELRIATPKGYEVNQKILDRALEFAKISGAKIVIGNDPIEAIKNATVVTTDTWVSMGQEDEKEKRVKDFTGYIVDTKLMSYANSNAIFLHCLPAYREYEVSEEVFEGPQSKVFEEAENRLHAQKGIMVWLDGKRND |
| NPA74209.1 | *Campylobacterota bacterium* | MRHFLTLNDFTKDEILEILNLAIEIKDELKSGNIKYRMKDKSLAMIFEKSSTRTRVSFEVGMTQLGGHALFLSSKDTQLGRGEPLKDTARVISRMVDMVMIRTYKQKDLEEFASYSKVPVINGLTDEYHPVQLMADYMTMMELGKDKEPKVAYIGDGNNMAHSWLMLASKLGFELRVATPKGYEVNDDILHTALQNAKVSGAKIVILNDPKEAVKGCDVVTTDTWASMGQEDEKAQRERDFQGYIVDSDLMRLAKDDAIFLHCLPAYRGQEVSEEVLESPQSVIFQEAENRLHAQKGIMVWLDKNRGDK |
| **ID** | **Species** | **Sequence**  **Table S3 (continued)** |
| MFA7084797.1 | *Arcobacteraceae bacterium* | MKHFLTLADFTKEELLEILELSLKIKTETKNKVYKDYMPKQVLGMIFEKSSTRTRVSFEAGIYQLGGIGLFLSSNDIQLGRGEPMNDTSRVITRMVDMVMIRTFEHAKLEEFTKHSQVPVINGLTDDFHPVQLIADYLTIMEAEKTENLIAAYIGDGNNLAHSWLMLASKMGFELRIATPKGCEVNPKFLQKALEFAKISGAKISVGNDPKEAVKNATVVTTDTWISMGQEDQKENKIKEFKGYIVDDSMMSLASKDAIFLHCLPAYRGYEVSESVFEGSQSRVFEEAENRLHAQKGIMVWLDGKRND |
| WP_104724632.1 | *Helicobacter cholecystus* | MKHFLSLKDFSKEEILEIISLAKHIKHDFKNNIPTPLMPQKILAMIFEKNSTRTRVSFEAGIYQLGGVGLFLSSNDIQLSRGEPIKDTARVISSMVDMIMIRTFEHSRLEEFASYCSVPVINGLSDSYHPVQLMADYLTMLECGISLEGEKPIVAYIGDGNNMAHSWLLLASKLGFELRIATPKNYEVDHKILALAMSFAQESGSKIITTHDPKEAIKGCNVVTTDTWISMGQEDQKNKKINDFKGFEVDKEMMHLAQSHAIFLHCLPAYRGYEVSEEVLEGTQSRVFLEAENRLHAQKGIMVYLYQNKEKLC |
| WP_112051905.1 | *Helicobacter monodelphidis* | MRHFLTLKDFSAKEIKEIVGLAIKIKKDLKNGKRESLLHGKTLAMIFEKNSTRTRVSFEAGIYQLGGIGLFLSNRDLQLGRGEDMKDTARVISRMVDMVVIRTDSHMRLEEFAEFSHVPVINGLSDSYHPTQLLADYLTMIECKKDKNPIVAYIGDGNNIAHSWLMLAAKMGFELRIATPSGYEPNISIYEDALEIAEESGAKIILSNDPKEAIKDATVVTTDTWTSMGQEEQKEQRLMDFKGFCIDTALFSKADPEAIFLHCLPAYRGQEVSDRVLEGERSAVFLEAENRLHIQKGIMVWLDQKRN |
| WP_200762641.1 | *Nitrosophilus alvini* | MKHFLTLRDFSKEEILEIIDLAISIKKEAKKGEFVPYLEGQTLAMIFEKSSTRTRVSFEVGIYQLGGIGLFLSKNDLQLGRGEPMKDTARVISRMVDMVMIRTYSQAKLEEFAKFSKVPVINGLTDEYHPVQLMADYMTMIEHRKENNPVVAYIGDGNNMAHSWLMLASKLGFELRIATPKGYEPDSKIVEDAFTFAKKSGAEILLTNDPKEAVRDADVVTTDVWASMGQEDEYEKRLKDFAGFMVDSTLMQLAKKDAIFLHCLPAHRGEEVSEEVLEGPQSVVFEEAENRLHAQKGIMVWLDRQRNKSIQE |
| **ID** | **Species** | **Sequence**  **Table S3 (continued)** |
| WP_353661910.1 | *Hydrogenimonas leucolamina* | MRHFLTLKDFTKEEILEMIDLGLRIKAEVKARRFTPYLEKQTLAMIFEKSSTRTRVSFETGIYQLGGMGLFLSSRDIQLGRGEPMKDTARVIGRMCDMVMIRTYSQSKLEEFARFSSVPVINGLTDEYHPVQLMADYMTMIEHGKADNPVVAYVGDGNNMAHSWLMLAAKLGFELRVATPKGYECNPDIVEDAFEIAKTSGATILFTDDPKEAVKGADVVTTDTWVSMGEEDEKEKRLRDFAGYTVDSGMMKLAKADAIFLHCLPAYRGMEVTEEVLEGPQSVVFDEAENRLHAQKGVMVWLDGKRNEA |
| MDD3324365.1 | *Sulfurospirillaceae bacterium* | MRHFLTLKDFSKEEILEIIDLAEKIKKKAKKGDFKHYLKDQTLGMIFEKSSTRTRVSFEVGIHQLGGKGLFLSSNDLQLGRGEPMKDTARVISRMVDMVMIRTYAQNTLEEFAEYSRVPVINGLSDSYHPVQLMADYLTMREYGKDKNPIVAYVGDGNNMAHSWLMLASKLGFELRIATPKGYEVDSTILEEALTFASSSGAVIKIGNDPKEAIKGANVVTTDTWVSMGQEGDKEKKVKDFKGYMVNVKMMSLAQKDAIFLHCLPAYRGYEVSEEVFESHADEIFDEAENRLHAQKGVMVWLDRNKEE |
| WP_286337826.1 | *Hydrogenimonas cancrithermarum* | MRHFLTLKDYTKEEILEIIELGLRIKAEVKAKRFTPYLEKQTLAMIFEKSSTRTRVSFETGIYQLGGMGLFLSSRDIQLGRGEPMKDTARVISRMCDMVMIRTYSQSKLEEFASFSNVPVINGLTDEYHPVQLMADYMTMMEHGKDRNPVVAYIGDGNNMAHSWLMLAAKLGFELRIATPKGYECNPEIVVDAMEFAQRSGAALLFTNDPEDAVAGADVVTTDTWVSMGDEEEKEKRLRDFAGYTVDEKMMKLAHADAIFLHCLPAYRGLEVTEDVLEGPQSVVFDEAENRLHAQKGVMVWLDGKRDEV |
| WP_099342348.1 | *Malaciobacter molluscorum* | MRHFLTLADYTKEEILEILELSRKIKKETKNRVFKDYMHKKTLGMIFEKSSTRTRVSFETGIYQLGGVGLFLSSNDIQLGRGEPMSDTSRVITRMVDMVMIRTFEQSKLEEFAKYSKVPVINGLTDEYHPVQLMADYLTIQEAGLDKNLIVAYVGDGNNMTHSWLNLAAKLGFELRIATPKGYEVNEDILVNALEMAKKSGAKIIITNDPKIAVKDATVVTTDTWVSMGQEEEKEQRIKDFNGYIVDKSMMELAQEKAIFLHCLPAYRGYEVSEEVMEGPQSLIFQEAENRLHAQKGVMVWLDRQRDK |
| **ID** | **Species** | **Sequence** |
| MCG3716244.1 | *Aliarcobacter butzleri* | PMSDTSRVISRMADMVMIRTFEQSKIEEFAKYSKVPVINGLTNEYHPVQLMADYMTIQEAGLEKDLVVAYVGDGNNMAHSWLNLAAKLGFELRIATPKGYEVDKNILNNALEMAKISGAKIIIGNDPKTAIKDSTVVTTDTWVSMGQEDEKEKRVKDFAGYMVDADMMKLAQDKAIFLHCLPAYRGYEVSEEVIESSQSLIFEEAENRLHAQKGVMVWLDRKRDEK |
| WP_138153647.1 | *Arcobacter arenosus* | MRHFLTLRDYTKEEILEILELAAKIKRETKNKVFKDYMPRNTLGMIFEKSSTRTRVSFEVGIYQLGGVGLFLSSNDIQLGRGEPMSDTSRVVSSMVDMVMIRTFAQSKIEEFAKYSKVPVINGLTDEYHPVQLMADYMTIQEAGLEKDLVVSYVGDGNNLAHSWLMLASKLGFELRIATPKGYEVNQNILDEALNFAKESGAKIIIGNDPKEACKDATVITTDTWVSMGQEDEKEKRLKDFDGYIVDKEMMKLGKPNAVFLHCLPAYRGYEVSEDVIEGEESLVFEEAENRLHAQKGVMVWLSRQINK |
| WP_345993281.1 | *Hydrocurvibacter sulfurireducens* | MKHFLTLKDFTKEEILEIINLGLEIKKDLKNGIHKDYMSKKTLAMIFEKSSTRTRVSFETGIYQLGGHGLFLSNRDIHLGRGEPVKDTARVISSMCDMVMIRTFEHSKIEEFSSYSKVPVINGLTDSYHPVQLLADYMTMIEYGVAENTVVAYIGDGNNMTHSWLMLASKLGFELRIATPKGYEVDSKVLEDAYAFAKKSGAKIFVSNDPKEAIRGANVVTTDTWASMGQESEKEQRIKDFKGFMVDDLMMCIADKNAKFLHCLPAYRGQEVSESVLEGHSDIVFDEAENRLHAQKGLMVWLDRQRGGSK |
