# Supplementary material for: Lactiplantibacillus plantarum AS21 Attenuates Sex‐Biased Glucose Intolerance in Aging Mice via Gut Mucosal Microbiota‐Derived Citrulline
Source: Aging Cell. 2026 Aug 13;25(8):e70662. doi: 10.1111/acel.70662 (PMC13473181; doi:10.1111/acel.70662)
Supplement: Supplementary file 18 [file ACEL-25-e70662-s017.docx]

**Supplementary Methods**

**1 Probiotic strain and preparation of probiotic powder**

*L. plantarum* AS21 was originally isolated from traditional Tibetan yak yogurt and is preserved in the Qinghai–Tibet Plateau Lactic Acid Bacteria Germplasm Bank. The strain has been officially deposited in the China General Microbiological Culture Collection Center (CGMCC No. 24968). To prepare the probiotic powder, bacterial cells were cultured in MRS broth and harvested via centrifugation (5,000 × *g*, 10 min, 4°C). The resulting pellet was resuspended in a sterile cryoprotectant solution (8% skimmed milk, 10% sucrose, 3% glycerol, 3% sorbitol), followed by lyophilization and mechanical grinding. Viable bacterial counts were determined by standard plate counting and expressed as colony-forming units per gram (CFU/g).

**2 Untargeted metabolomic profiling**

Serum samples obtained from the natural aging model were subjected to untargeted metabolomic analysis (Wekemo Tech, Shenzhen, China). Briefly, the serum samples were mixed with pre-chilled 80% methanol (1:4, v/v), incubated on ice for 5 min, and centrifuged (15,000 × *g* for 20 min at 4°C). The resulting supernatant was diluted to a final concentration of 53% methanol using LC-MS grade water and centrifuged again. The final clarified supernatant was collected for LC–MS/MS analysis.

UHPLC–MS/MS analysis was performed using a Vanquish UHPLC system coupled to an Orbitrap Q Exactive™ HF mass spectrometer (Thermo Fisher Scientific, Germany). Chromatographic separation was achieved on a Hypersil Gold column (100 × 2.1 mm, 1.9 μm) at a constant flow rate of 0.2 mL/min, utilizing water containing 0.1% formic acid (Mobile Phase A) and pure methanol (Mobile Phase B). The gradient elution profile was programmed as follows: 2% B (0–1.5 min), 2–85% B (1.5–4.5 min), 85–100% B (4.5–10.5 min), 100% B (10.5–10.6 min), followed by re-equilibration at 2% B (10.6–12 min). The mass spectrometer was operated in a positive/negative ion switching mode with the following parameters: spray voltage 3.5 kV, capillary temperature 320°C, sheath gas flow rate of 35 arb, auxiliary gas flow rate of 10 arb, S-lens RF level of 60, and an auxiliary gas heater temperature of 350°C.

Raw UHPLC–MS/MS data were processed utilizing Compound Discoverer 3.3 software (Thermo Fisher Scientific) for peak alignment, peak detection, and quantification. Metabolites were identified by matching spectral features against the mzCloud, mzVault, and MassList databases, and were further annotated using the KEGG and HMDB databases. Differentially abundant metabolites were determined employing a Student’s t-test with false discovery rate (FDR) correction. Metabolites satisfying the criteria of |log₂ fold change| > 0.58 and FDR-adjusted *p* < 0.05 were considered significantly altered.

**3 Metagenomic sequencing and analysis of colonic mucosal microbiota**

Colon tissues were opened longitudinally and gently rinsed with PBS, after which the mucosal layer was carefully scraped off. Microbial genomic DNA was extracted utilizing a modified CTAB–phenol–chloroform method. Sequencing libraries were constructed using the NEBNext Ultra DNA Library Prep Kit and sequenced on an Illumina NovaSeq 6000 platform to generate approximately 10 Gb paired-end reads (150 bp) per sample.

Raw sequencing reads were quality-filtered using Trimmomatic (v0.39). Host DNA sequences were deleted by mapping the quality-controlled reads to the mouse reference genome (GRCm39) using KneadData in combination with Bowtie2. Taxonomic profiling was performed utilizing Kraken2, and relative abundances were subsequently estimated using Bracken. For functional analysis, the reads were *de novo* assembled using MEGAHIT, followed by gene prediction with Prodigal. A non-redundant gene catalog was then constructed utilizing CD-HIT. Gene abundance was estimated using Salmon and normalized to account for differences in sequencing depth. Functional annotation was performed against the EggNOG and CAZy databases.

Alpha diversity was evaluated using Chao1, Observed Features, Shannon, and Simpson indices. Beta diversity was assessed based on Bray-Curtis distances and visualized via principal coordinate analysis (PCoA), and statistical significance determined utilizing PERMANOVA. Differentially abundant taxa between groups were identified using the Wilcoxon rank-sum test.

**4 Quantification of metabolites in co-culture supernatants**

Amino acid-related metabolites were quantified via LC–MS/MS (LC-Bio Technologies, Hangzhou, China). Culture supernatants (100 μL) were mixed with 300 μL of pre-chilled methanol containing isotope-labeled internal standards, sonicated on ice, and incubated at −80°C to facilitate proteins precipitation. Following centrifugation, the clarified supernatants were analyzed using an Agilent 1290 UHPLC system coupled to an Agilent 6545 Q-TOF mass spectrometer (Agilent Technologies, USA). Metabolites were definitively identified based on accurate mass, retention time, and MS/MS fragmentation patterns, and subsequently quantified relative to the internal standards.

**5 Systematic reviews of clinical studies on citrulline and glycemic outcomes**

A systematic literature search was performed across the PubMed and Web of Science Core Collection databases to identify clinical studies evaluating the effects of citrulline on glycemic outcomes. The search was restricted to publications dated between January 1, 2015, and January 16, 2026. Search strategies employed combinations of “L-citrulline” or “citrulline” with terms related to prediabetes or type 2 diabetes (prediabetes, pre-diabetes, impaired glucose tolerance, impaired fasting glucose, insulin resistance, type 2 diabetes) and glycemic outcomes (glucose, blood glucose, glucose tolerance, HbA1c, insulin sensitivity, HOMA-IR). The search was limited to English-language articles. Retrieved records were imported into EndNote software, where duplicate entries were removed. Study selection was conducted in strict accordance with the Preferred Reporting Items for Systematic Reviews and Meta-Analyses (PRISMA) 2020 guidelines (Page et al. 2021). Initial screening based on titles and abstracts was followed by a comprehensive full-text evaluation of potentially eligible articles. The screening process was performed manually by a single independent investigator. To be eligible for inclusion, studies had to be human clinical trials that involved a citrulline intervention and reported glycemic-related outcomes. Conversely, animal studies, *in vitro* experiments, review articles, and studies lacking relevant endpoints were excluded. Ultimately, five clinical studies met all inclusion criteria, the details of which are summarized in Table S1. Notably, no studies specifically investigating citrulline intervention in prediabetic cohorts were identified. Given the limited number of eligible studies, a quantitative meta-analysis was not feasible.

**Reference**

Page, M. J., McKenzie, J. E., Bossuyt, P. M., et al. 2021. "The PRISMA 2020 statement: an updated guideline for reporting systematic reviews." *Bmj* 372, n71. https://doi.org/10.1136/bmj.n71.
